# Structure of the Arginine Methyltransferase PRMT5-MEP50 Reveals a Mechanism for Substrate Specificity

**DOI:** 10.1371/journal.pone.0057008

**Published:** 2013-02-25

**Authors:** Meng-Chiao Ho, Carola Wilczek, Jeffrey B. Bonanno, Li Xing, Janina Seznec, Tsutomu Matsui, Lester G. Carter, Takashi Onikubo, P. Rajesh Kumar, Man K. Chan, Michael Brenowitz, R. Holland Cheng, Ulf Reimer, Steven C. Almo, David Shechter

**Affiliations:** 1 Department of Biochemistry, Albert Einstein College of Medicine of Yeshiva University, Bronx, New York, United States of America; 2 Department of Physiology and Biophysics, Albert Einstein College of Medicine of Yeshiva University, Bronx, New York, United States of America; 3 Institute of Biological Chemistry, Academia Sinica, Nankang, Taipei, Taiwan; 4 JPT Peptide Technologies, Berlin, Germany; 5 Stanford Synchrotron Radiation Lightsource, SLAC National Accelerator Laboratory, Menlo Park, California, United States of America; 6 Department of Molecular and Cellular Biology, University of California Davis, Davis, California, United States of America; St. Georges University of London, United Kingdom

## Abstract

The arginine methyltransferase PRMT5-MEP50 is required for embryogenesis and is misregulated in many cancers. PRMT5 targets a wide variety of substrates, including histone proteins involved in specifying an epigenetic code. However, the mechanism by which PRMT5 utilizes MEP50 to discriminate substrates and to specifically methylate target arginines is unclear. To test a model in which MEP50 is critical for substrate recognition and orientation, we determined the crystal structure of Xenopus laevis PRMT5-MEP50 complexed with S-adenosylhomocysteine (SAH). PRMT5-MEP50 forms an unusual tetramer of heterodimers with substantial surface negative charge. MEP50 is required for PRMT5-catalyzed histone H2A and H4 methyltransferase activity and binds substrates independently. The PRMT5 catalytic site is oriented towards the cross-dimer paired MEP50. Histone peptide arrays and solution assays demonstrate that PRMT5-MEP50 activity is inhibited by substrate phosphorylation and enhanced by substrate acetylation. Electron microscopy and reconstruction showed substrate centered on MEP50. These data support a mechanism in which MEP50 binds substrate and stimulates PRMT5 activity modulated by substrate post-translational modifications.

## Introduction

The family of protein arginine methyltransferases (PRMTs) in metazoans includes at least 10 proteins with diverse roles [1]. The majority of these enzymes are Type I enzymes that are capable of mono- and asymmetric dimethylation of arginine, with S-adenosylmethionine (SAM) as the methyl donor. PRMT5 is a Type II enzyme, capable of mono- and symmetric dimethylation [2]–[4]. PRMT5 methylates histones H2A and H4 on Arg3 [5], histone H3 on Arg2 [6] and Arg8, and many other proteins [7]. PRMT5 is required for stem cell maintenance and developmental growth in *Planaria* [8], in mouse embryonic and induced pluripotent stem cells [9], [10], and is required for initiation of differentiation in myogenesis [11]. PRMT5 prevents keratinocyte differentiation [12] and may be responsible for stem cell maintenance in germ cell tumors [13].

PRMTs and histone arginine methylation are heavily enriched in eggs and early embryos of metazoans [5], [9], [14]. We previously showed that PRMT5-MEP50 methylates histones H2A and H4 and the histone chaperone Nucleoplasmin in *Xenopus laevis* eggs [5]. Furthermore, PRMT5 regulates transcription via histone methylation, specifically down-regulating transcription of ribosomal genes, cyclin E, Rb, and other genes [15]–[17]. PRMT5 partners with many protein cofactors, including Blimp1 [14], RioK1 [18], pICLn [19], MBD/NuRD [20], and MEP50 [21]. MEP50, a WD-40 repeat protein, is its most common partner and likely present in every PRMT5-containing complex *in vivo* [1]. Recent reports demonstrated that phosphorylation of PRMT5 by mutant Jak2 kinase and of MEP50 by Cdk4 altered the activity and targeting of the PRMT5 enzyme leading to tumorigenesis [22], [23]. Insight into the location of these phosphorylation sites would illuminate the potential oncogenic mechanisms promoted by these aberrant kinase targets. Furthermore, how PRMT5 interacts with protein cofactors to alter its activity and gain substrate specificity is unclear.

PRMT5 forms high molecular weight complexes [24]. *X. laevis* PRMT5-MEP50 (*Xl*PRMT5-MEP50) forms an assembly larger than expected for a simple heterodimer pair [5]. PRMT1, PRMT3 and PRMT4 (CARM1) dimerize using a dimerization arm located at the C-terminus [25]. The structure of *C. elegans* PRMT5 (*Ce*PRMT5) exhibited a head-to-tail dimer, with the N-terminus of one PRMT5 molecule contacting the C-terminus of its interacting molecule [26]. However, *Ce*PRMT5 is only 29% identical to the *Xenopus* protein, *C. elegans* does not contain a MEP50 ortholog, and no cofactors for *Ce*PRMT5 have been identified.

Here we report the structure of the full-length *X. laevis* PRMT5-MEP50 complex crystallized in the presence of S-adenosylhomocysteine (SAH), the byproduct of the methylation reaction. PRMT5-MEP50 forms an unusual tetramer of heterodimers with four copies each of PRMT5 and MEP50. We demonstrate that PRMT5-MEP50 activity is modulated by substrate post-translational modifications and that MEP50 is required for stimulating PRMT5 activity. Our data suggest that a primary function of MEP50 is to bind and orient the arginine-containing substrate to the PRMT5 catalytic site. Furthermore, PRMT5 enzyme turnover may be modulated by charge-shifting substrate post-translational modifications.

While this manuscript was under review, the highly similar human PRMT5-MEP50 structure was independently reported [27].

## Results

### PRMT5 forms an Unusual Dimer of Dimers

**Figure 1:**
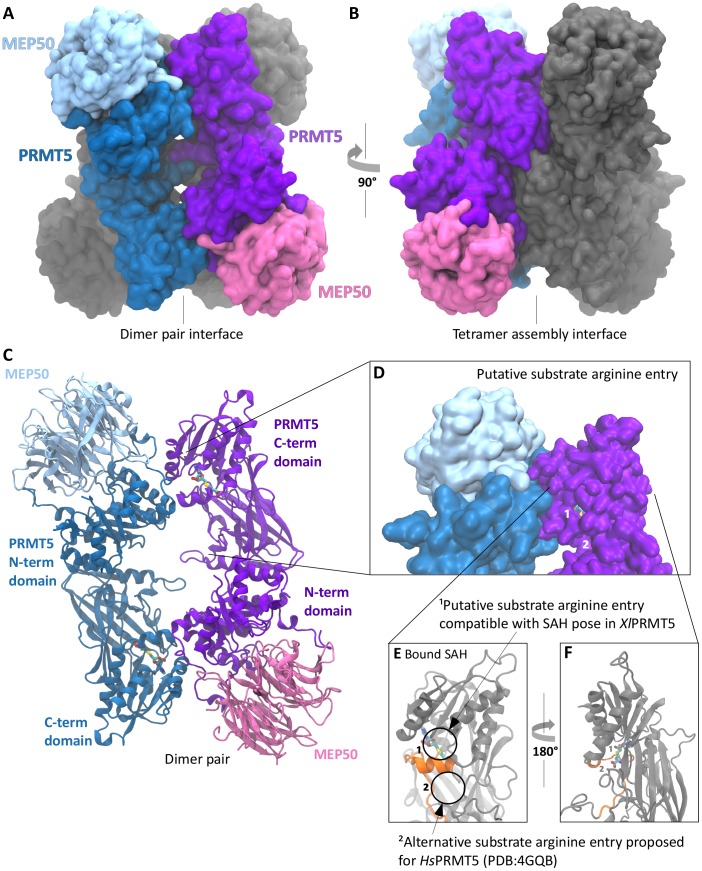
PRMT5-MEP50 overall structure. **A.** PRMT5-MEP50 tetrameric surface-filled model. The dimer of PRMT5 molecules is arranged in a head-to-tail form (dark blue and purple). MEP50 is bound to the N-terminus of each PRMT5 molecule on the oblong face of the WD40 beta propeller (light blue and pink). The molecule has 2-fold rotational symmetry through the axis perpendicular to the page. **B.** PRMT5-MEP50 tetramer rotated 90° with the tetramer pair of PRMT5-MEP50 heterodimers shown in gray. **C.** PRMT5-MEP50 dimer pair shown in cartoon form with the bound SAH visible. N-terminal and C-terminal domains are indicated. **D.** A surface view of the area around the SAH-bound active site of one PRMT5 molecule (purple). The cross-dimer bound MEP50 is shown in light blue, with the dimer paired PRMT5 in dark blue. The putative substrate arginine insertion pocket is circled, with the SAH visible (sulfur in yellow). **E.** Cartoon representation of PRMT5 C-terminal domain (gray) with the active SAH bound, shown from the solvent accessible face with our proposed substrate entry pocket circled^1^. The helix and loop colored orange (residues 303 to 324) is isostructural with a domain that is unstructured (no electron density) in the absence of SAH in 3UA4. The substrate arginine entry in PDB:4GQB is circled^2^. **F.** The N-terminal active domain rotated 180° to show the constraining beta sheets.

We crystallized *Xenopus* PRMT5-MEP50 complex in the presence of SAH (Table 1). The 3.0 Å structure revealed that PRMT5 forms a tetramer (a dimer of dimers with *D2* symmetry). Crystallographic symmetry results in an *Xl*PRMT5 dimer similar to those observed in all reported PRMT structures, including *Ce*PRMT5 (Figure 1a and 1b). We will refer to this association as the “dimer pair” and the corresponding interface as the “dimer interface” throughout. The two PRMT5 molecules in the asymmetric unit form a previously uncharacterized interface which results in the observed tetrameric assembly. We will refer to this association as the “tetramer dimer” and the related interface as the “tetramer interface”. A central cavity of approximately 30 Å in diameter is evident on one face of the tetramer (Figure 1a). MEP50 is not directly involved in the PRMT5 oligomeric interactions.

**Table 1:** Data collection and refinement statistics for PRMT5-MEP50.

	*Xenopus laevis* PRMT5-MEP50
**Data collection**	4G56
Space group	P2_1_2_1_2
Cell dimensions	
* a*, *b*, *c* (Å)	181.86, 101.94, 125.68
α, β, γ (°)	90.0, 90.0,90.0
Resolution (Å)	50.00–2.95 (30.06–2.95)*
*R*_sym_	15.0 (89.8)
*I/*σ*I*	12.9 (2)
Completeness (%)	99.9 (99.9)
Redundancy	6.3 (6.0)
**Refinement**	
Resolution (Å)	50.00-3.0
No. reflections	49885
*R*_work_/*R*_free_	21.8/27.5
No. atoms:	
Protein	14413
Ligand/ion	52
Water	9
*B*-factors:	
Protein	65.3
Ligand/ion	47.2
Water	34.1
RMS deviations:	
Bond lengths (Å)	0.002
Bond angles (°)	0.566
**Ramachandran analysis**	
Favored region	94.5%
Allowed region	5.1%
Disallowed region	0.4%

* Numbers in parentheses are for highest-resolution shell.

The overall organization of *X. laevis* PRMT5 (*Xl*PRMT5) is similar to *Ce*PRMT5 (PDB:3UA3). Discrete N- and C-terminal domains are connected by an unstructured loop (Figure S1a). *Xl*PRMT5 and *Ce*PRMT5 C-terminal β-barrel and Rossmann folds exhibit an average root-mean-square deviation (RMSD) of 1.1 Å (317 aligned residues). The long dimerization arm in *Ce*PRMT5 is a small loop in the *Xenopus* structure (Figure S1a). The N-terminal domain forms a TIM barrel with two protruding loops not present in the *C. elegans* structure. These orthologs show significant structural similarity, with an average RMSD of 1.5 Å (227 aligned residues). We observed interpretable electron density for a segment connecting the N-terminal TIM barrel and the C-terminal Rossmann fold of *Xl*PRMT5. Therefore our assignment of the orientation of the arrangement of the N-to-C terminal domains differed from that assigned to the *C. elegans* PRMT5 (PDB:3UA3). Sequences of *Xenopus* and human PRMT5 N-terminal domains are highly similar with only minor amino acid changes (Figure S2).

### SAH Interacting Residues and the Catalytic Site

We readily identified SAH in a conserved catalytic site. Difference Fourier synthesis (F_o_–F_c_ contoured at 3σ) clearly showed bound SAH (Figure S3). The relative pose of the adenosyl moiety of SAH in *Xl*PRMT5 is similar to other PRMTs. The relative pose of the homocysteine moiety in *Xl*PRMT5 is flipped 98° towards Trp575 compared with *Ce*PRMT5 (Figures S3, S4a,b and S8). The prominent electron density assigned to the SAH sulfur in the *Xenopus* structure allows for confident placement adjacent to a small channel that connects the PRMT5 catalytic site with bulk solvent. This model suggests that the methyl donor on SAM would also be facing solvent and therefore this channel could support the entry of the substrate arginine guanidinium group into the catalytic site. This narrow catalytic site entry pocket on the outer face of PRMT5 is adjacent to the N-terminus of its dimer-paired PRMT5 and MEP50, and distal to its directly-associated MEP50 (Figure 1d,e, circled pocket 1). However, this channel is too small to support SAM exchange, perhaps requiring movement of a loop (Figure 1e, orange, residues 303 to 324) shown to be disordered in the absence of SAH in the *C. elegans* PRMT5 structure (αA helix in 3UA4).

The invariant glutamic acid residues (Glu431 and Glu440) in the “double-E” loop are hydrogen bonded to SAH (Figure S2). The PRMT5-specific phenylalanine (F323 in this structure) that is required for symmetric arginine dimethylation is positioned in the catalytic site along the αA helix [26]. The location of the alternative substrate arginine entry demonstrated for *Hs*PRMT5 is shown (Figure 1e,f, circled pocket 2) [28].

### MEP50 Structure

MEP50 adopts a seven-bladed toroidal WD40 repeat (Figure 1c and Figure S1b). The last blade contains three β-strands and lacks the “Velcro” closure typical of WD-repeat proteins [29]. The poorly conserved and disordered N-terminus of MEP50 may fold back to form a fourth β-strand to complete the expected WD-repeat. MEP50 also has an unusual extension of one of its β-sheet blades that contains a highly conserved arginine residue (R42) on its tip. Four MEP50 molecules were bound to the four PRMT5 molecules as heterodimers (Figure 1a and 1c).

We compared MEP50 with WDR5 (PDB:2H68), a WD-repeat protein that recognizes histone H3 tails for the MLL lysine methyltransferase [30]. These proteins are similar, with an average RMSD of 2.0 Å (264 aligned residues). WDR5 is a basic protein, with a calculated pI of 8.4 (human), while MEP50 is highly acidic, with a theoretical pI of 5.1 (*Xenopus*). PRMT5 is also an acidic protein with a theoretical pI of 5.8. The electrostatic surface potential of the PRMT5-MEP50 complex reveals an extended negatively charged surface, consistent with recruitment of basic substrates such as histone tails (Figure S1c).

### XlPRMT5-MEP50 Forms a Tetramer of Heterodimers

**Figure 2:**
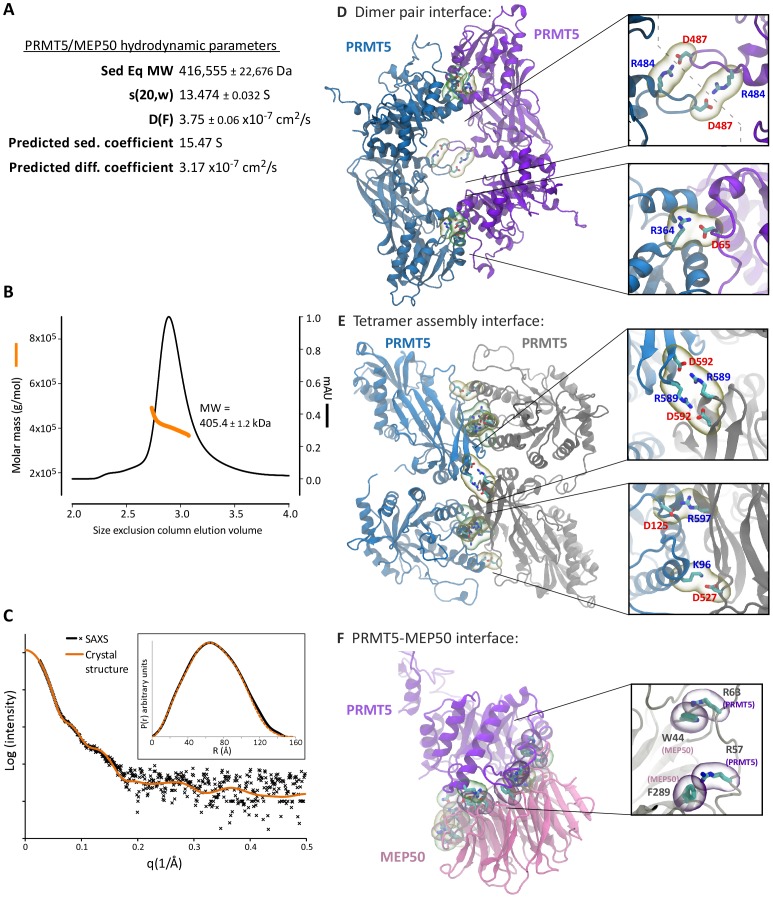
Hydrodynamic studies demonstrate that PRMT5-MEP50 forms a higher order tetrameric structure. **A.** Analytical equilibrium and sedimentation velocity centrifugation studies gave a molecular weight and sedimentation and diffusion coefficients as shown. Hydropro calculated sedimentation and diffusion coefficients from the structure are also shown. **B.** Size-exclusion chromatography multi-angle light scattering profile, with the protein elution UV profile shown in black and the calculated molar mass from the Rayleigh plot shown in orange. **C.** Small-angle x-ray scattering curve showing that the solution scattering data matches well with the crystal structure. Inset: Pairwise distribution function P(r) compared with crystal structure. **D.** The PRMT5 dimer interface is illustrated in cartoon form. One PRMT5 is in blue and the paired molecule is in purple, arranged with the N-terminal domain paired with the C-terminal domain of the neighboring protein. Salt bridges are in yellow bubbles and hydrogen bonds are in green bubbles. The insets highlight salt bridges between R484 and D487 of paired PRMT5 on the dimerization arms and between R364 and D65 on the head-to-tail interface. Gray dashed line shows the boundary between molecules. **E.** The PRMT5 tetramer interface is shown, with one PRMT5 colored blue and the paired molecule in gray. Substantial salt-bridges (yellow bubbles) and hydrogen bonds (green bubbles) are shown. The insets highlight salt bridges between R589-D592 and between D125-R597 and K96-D527 of paired PRMT5 molecules. Gray dashed line shows the boundary between molecules. **F.** The PRMT5-MEP50 interface is shown, with PRMT5 in purple and MEP50 in pink. Substantial specific contacts are shown, with the inset illustrating cation-pi interactions between R63 and R57 of PRMT5 with W44 and F289 of MEP50, respectively (purple bubbles).

We conducted several independent studies to confirm that the oligomeric state observed in the crystal structure is present in solution. Sedimentation equilibrium ultracentrifugation demonstrated a mass consistent with a tetramer of heterodimers: 4 each of PRMT5 and MEP50 (predicted mass of the recombinant proteins is 454 kDa). Experiments performed in 2 M NaCl exhibited no change in mass, consistent with a stable tetramer. Sedimentation velocity experiments revealed sedimentation and diffusion coefficients consistent with both the equilibrium value as well as the Hydropro [31] predicted coefficients calculated from the crystallographic structure (Figure 2a). Size-exclusion chromatography coupled with multi-angle light scattering yielded a molecular weight of the *Xl*PRMT5-MEP50 assembly of 405.4 kDa, also consistent with a tetrameric complex (Figure 2b). Finally, FPLC-SAXS experiments were performed to obtain a monodispersed sample for in line small-angle X-ray scattering (SAXS) measurements. *Xl*PRMT5-MEP50 applied to a gel-filtration column eluted as a single peak and scattering profiles over the peak were used for further analysis. The SAXS data fit the simulated scattering curve of the structure generated with CRYSOL [32] (Figure 2c). Importantly, there was considerable correlation between the experimentally determined solution and structure-calculated pairwise distribution function P(r) (Figure 2c, inset). Together, these independent measures support the *Xl*PRMT5-MEP50 tetramer observed as the relevant assembly in solution.

### PRMT5 Dimerizes Head-to-tail within the Tetramer

There are numerous points of interaction within the PRMT5 dimer pair and the tetramer interface, including contributions from the N-terminal TIM barrel domain as well as in the Rossmann fold and β-barrel in the C-terminus (listed in Figure S4). The dimer pair arrangement is identical to other PRMTs, with the N-terminus of one molecule making substantial contacts with the C-terminus of the other, including reciprocal salt bridges between R364 and D65. An arm extends across the central hole between the dimers, forming two reciprocal salt bridges between R484 and D487 (Figure 2d and insets). The tetramer interface includes multiple salt bridges, including reciprocal interactions between R589 and D592 as well as interactions between D125 and R597 and D527 and K96 (Figure 2e and insets). All the residues involved in dimerization are evolutionarily conserved, while the residues involved in tetramerization are conserved in metazoa (Figure S2).

### A Tight Interface Connects PRMT5 and MEP50

MEP50 forms a seven-bladed beta-propeller and utilizes a large surface on one end to interact with the N-terminus of a single PRMT5 monomer. Residues 39–44 of MEP50 form a short β-hairpin and protrude to interact with residues 17–20, 40–45 and 61–63 of PRMT5 (Figure S1, S4). A loop from residues 152 to 178 of PRMT5 protrudes out and interacts with residues on the second and third beta propeller blades of MEP50, including residues 154–158, 181–185, and 191–195. *Xl*PRMT5-MEP50 interactions include salt bridges, cation-pi interactions, and many hydrogen bonds (Figure S4e,f). PRMT5 residues R57 and R63 interact with MEP50 F289 and W44, respectively, by cation-pi interactions and are conserved among vertebrates (Figure 2f). W44 is found on the MEP50 insertion finger while F289 is found next to the PRMT5 interaction loop. None of the PRMT5-MEP50 interactions are directly involved in PRMT5 oligomerization.

### PRMT5-MEP50 Activity is Influenced by Substrate Complexes

**Figure 3:**
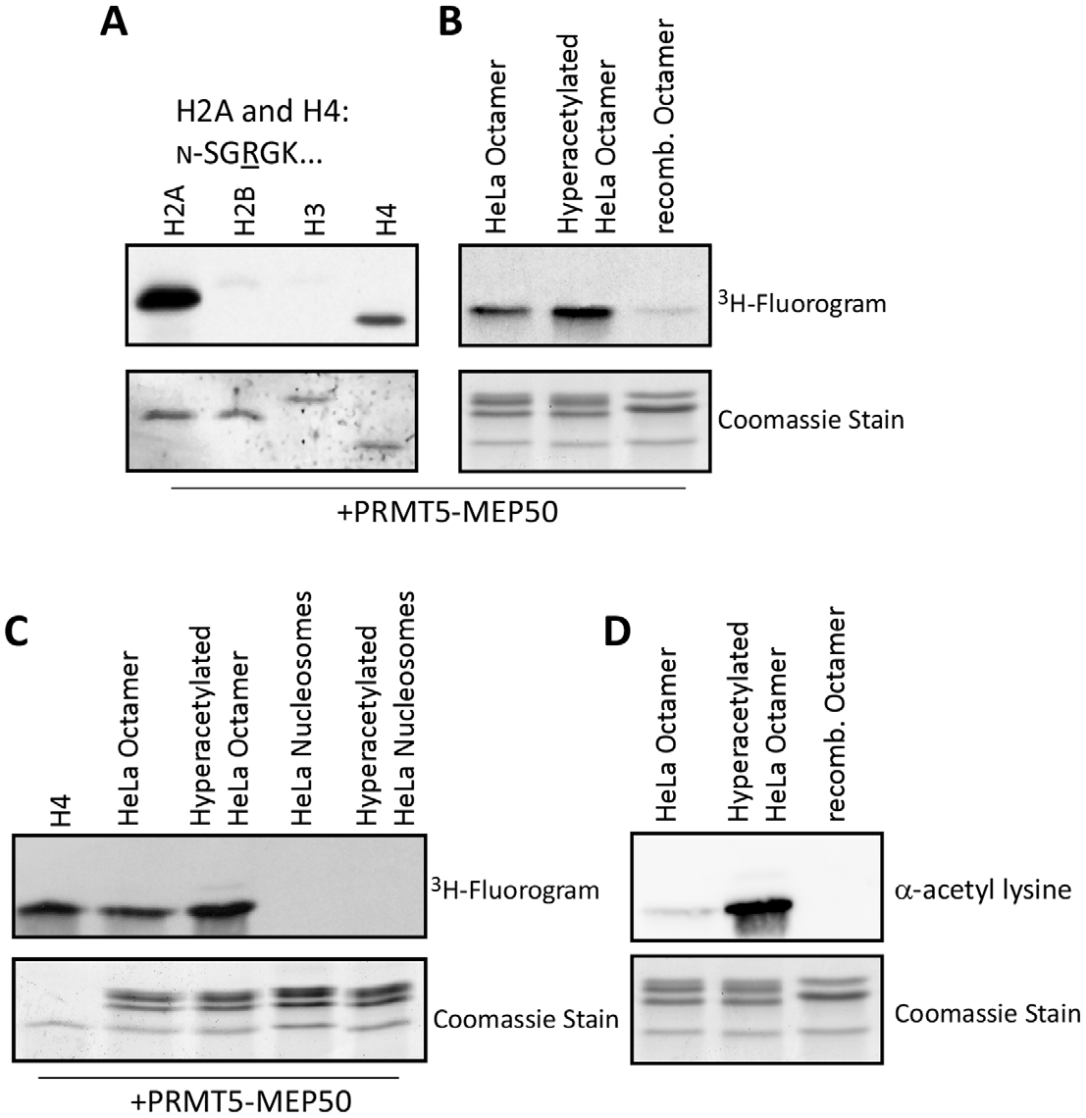
PRMT5-MEP50 histone methyltransferase activity is modulated by substrate complexes and acetylation state. Recombinant PRMT5-MEP50 (50 nM tetramer) was used in methyltransferase assays with histone substrates and ^3^H-SAM as indicated; NaCl was maintained at a final 250 mM concentration. For each experiment, the fluorogram is on the top panel, Coomassie-stain on the bottom: **A.** Full-length recombinant *Xenopus laevis* histones H2A, H2B, H3, and H4. **B.** Histone octamers purified from HeLa cells, octamers purified from butyrate-treated HeLa cells, and recombinant octamers. **C.** Recombinant H4, octamers and nucleosomes from HeLa cells and butyrate-treated HeLa cells. **D.** Immunoblot with anti-acetyl lysine antibody on octamers from untreated and butyrate-treated HeLa cells and recombinant octamers.

PRMT5-MEP50 methylates histones H2A, and H4 (Figure 3a). PRMT5-MEP50 activity towards H4, but not H2A, was stimulated on octamers extracted from Hela cells. Activity was further stimulated by hyperacetylated octamers extracted from butyrate-treated HeLa cells. The enzyme complex poorly methylated recombinant histone octamers. All substrate complexes were rapidly diluted into the reaction at constant final 250 mM salt concentration (Figure 3b, 3c). PRMT5-MEP50 did not methylate mononucleosomes isolated from untreated or butyrate-treated HeLa cells (Figure 3c). The increased histone acetylation on butyrate-treated HeLa histones was confirmed by immunoblotting with an anti-acetyllysine antibody (Figure 3d).

**Figure 4:**
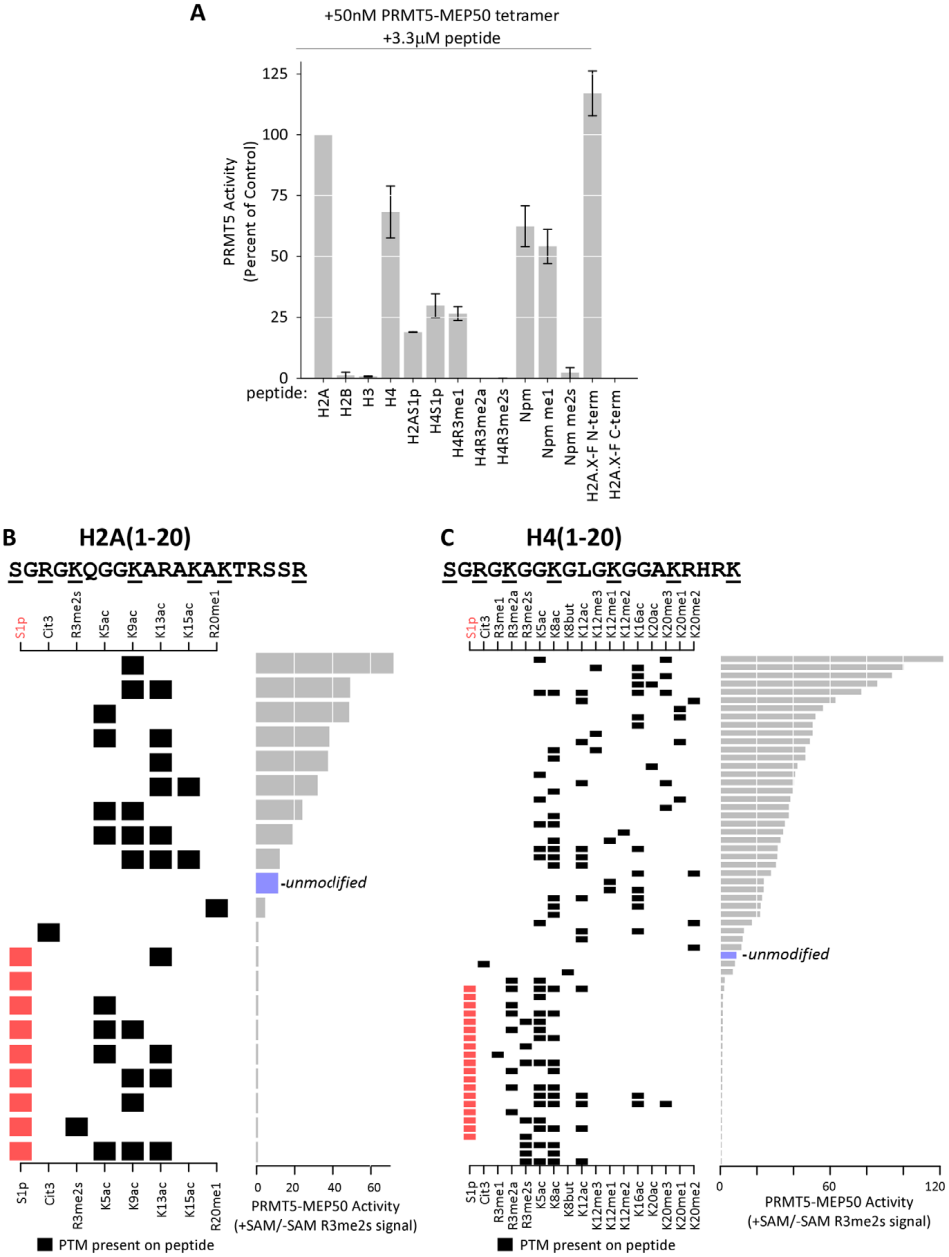
PRMT5-MEP50 histone methyltransferase activity is modulated by substrate PTMs. **A.** Recombinant PRMT5-MEP50 (50 nM tetramer) was used in duplicate solution methyltransferase assays with 3.3 µM histone and Nucleoplasmin peptide substrates (20mers) and ^3^H-SAM as indicated. Histone peptides containing modifications are as indicated: S1ph = Serine 1 phosphorylation; R3me1 and R3me2 = Arginine 3 methylation; Npm me1 and me2s = Arginine 187 methylation. Data shown as percent of H2A(1–20) activity. **B** and **C.** High-density histone peptide arrays incubated with PRMT5-MEP50 in the presence or absence of SAM. The arrays were probed with anti-methylarginine antibodies and background (-SAM) was subtracted from the fluorescence signal. Data from N-terminal H2A (B) and H4 (C) peptides are shown. The sequence of H2A and H4 (1–20) are illustrated at the top. Each row represents a discrete peptide. The left panel shows individual modifications present on each peptide, with a black box indicating its presence and white illustrating its absence. The histogram on the right panel shows the relative activity (ratio of antibody signal +SAM vs. –SAM) on each peptide. The signal on the unmodified 1–20 peptide is indicated (blue). Inhibition by Ser1 phosphorylation is indicated in red.

To further test the role of substrate PTMs in modulating PRMT5 activity we performed histone methyltransferase assays on modified peptides (Figure 4). These assays confirmed that *Xl*PRMT5-MEP50 specifically methylated H2A and H4 on R3; it did not methylate histone H3. Phosphorylation of Ser1 on H2A and H4 (S1ph) greatly reduced the activity of PRMT5-MEP50. The enzyme also methylated the known substrate Nucleoplasmin C-terminal tail peptide and the histone H2A.X-F N-terminal, but not C-terminal peptide (Figure 4a) [5]. We then probed its activity on histone peptides immobilized in high-density arrays. H2A and H4 activity profiles were generated from H2A/H4R3me2s antibody recognition of modified peptides following incubation of PRMT-MEP50 on the array in the presence and absence of SAM (Figure 4b,c). The R3me2s antibody retained recognition of methylated arginine in the presence of S1 phosphorylation and neighboring lysine acetylation on untreated peptide arrays (Figure S5). These data demonstrated that the presence of S1ph eliminated methyltransferase activity towards the peptides. Conversion of R3me1 to R3me2s was not observed as R3me1 was not present on the array in the absence of S1 phosphorylation. Conversion of R3me1 to R3me2s was observed on the R3me1 containing peptide in the solution assay (Figure 4a). Loss of activity on citrulline-3 (Cit3) containing peptides confirmed that the major H2A and H4 target of PRMT5-MEP50 is arginine 3. Site-specific combinations of lysine acetylation and lysine methylation led to substantially increased activity, consistent with the solution assay on HeLa histones (Figure 3b,c).

### MEP50 Stimulates PRMT5 Activity

**Figure 5:**
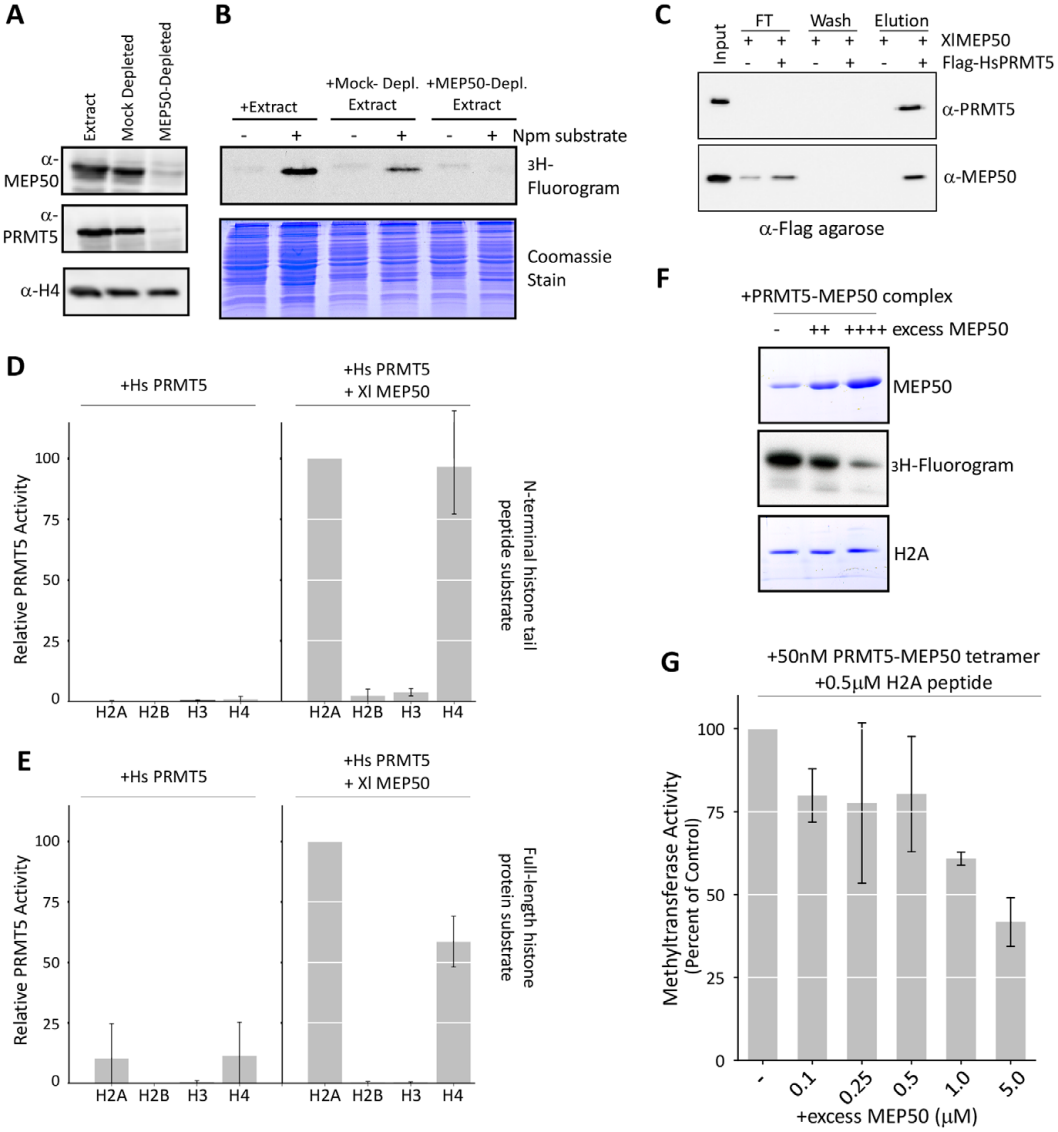
MEP50 is required for PRMT5 histone methyltransferase activity. **A.** *Xenopus* cell-free egg extract was mock-depleted or MEP50-depleted and an aliquot was blotted for PRMT5, MEP50, and H4 (as a depletion control). **B.** Egg extract, mock-depleted, or MEP50-depleted egg extract was incubated with ^3^H-SAM in the absence or presence of excess recombinant Nucleoplasmin (Npm); endogenous Npm is already methylated. The reaction was run on a gel and exposed to film. **C.** Recombinant *Xl*MEP50 was incubated in the presence or absence of Flag-*Hs*PRMT5 (both at 50 nM) and applied to anti-Flag resin. The flow-through (FT), final wash, and eluent were immunoblotted for PRMT5 and MEP50. **D.** Recombinant Flag-*Hs*PRMT5 (220 nM) was used in triplicate solution methyltransferase assays with histones H2A, H2B, H3, and H4 tail peptides (42 µM) in the absence (left) or presence (right) of *Xl*MEP50 (220 nM). **E.** Recombinant Flag-*Hs*PRMT5 (220 nM) was used in triplicate solution methyltransferase assays with full-length core histones H2A, H2B, H3, and H4 (6 µM) in the absence (left) or presence (right) of *Xl*MEP50 (220 nM). **F.** Recombinant *Xl*PRMT5-MEP50 was used in a methyltransferase assay with constant full-length histone H2A as a substrate. Increasing doses of excess *Xl*MEP50 were added to the reactions and the results were run on a gel, Coomassie stained, and exposed to film. **G.** Recombinant *Xl*PRMT5-MEP50 (50 nM tetramer) was used in solution methyltransferase assays with 0.5 µM histone H2A (1–20) peptide. Excess *Xl*MEP50 protein was titrated in to final concentrations between 0.1 and 5.0 µM and methyltransferase activity was assayed. The plot represents the diminution of activity in the presence of excess *Xl*MEP50.

We aimed to test the role of MEP50 in PRMT5 methyltransferase activity. First, we demonstrated that immunodepletion of MEP50 from *Xenopus* cell-free egg extract commensurately depleted PRMT5 and eliminated methyltransferase activity towards Nucleoplasmin, a known PRMT5 target, compared to mock-depleted extract (Figure 5a, b). This confirmed the *in vivo* stoichiometric relationship between MEP50 and PRMT5.

*Xenopus* PRMT5 was insoluble when expressed alone in bacteria and in insect cells and human MEP50 was also insoluble when expressed alone in bacteria (*unpublished observations*). Human PRMT5 (Flag-*Hs*PRMT5), 84% identical to the *Xenopus* protein (Figure S2a), was soluble when expressed in 293 cells, and importantly did not contain complexed MEP50 (Figure S6). To demonstrate that human PRMT5 forms a complex with *X. laevis* MEP50 (*Xl*MEP50), we co-purified the proteins on anti-Flag resin. *Xl*MEP50 specifically eluted with Flag-*Hs*PRMT5 (Figure 5c).

We then probed the methyltransferase activity of Flag-*Hs*PRMT5 and Flag-*Hs*PRMT5-*Xl*MEP50. *Hs*PRMT5 alone had negligible activity towards histone peptides or full-length protein under our experimental setup (Figure 5d,e). Activity towards H2A and H4 peptides and full-length protein was dramatically stimulated upon addition of *Xl*MEP50 (Figure 5d,e). We complemented this observation by adding excess MEP50 to a methyltransferase reaction with *Xl*PRMT5-MEP50 and H2A peptide or full-length protein. Excess *Xl*MEP50 inhibited the methyltransferase activity, consistent with MEP50 acting to sequester substrate from the enzyme (Figure 5f,g).

### PRMT5 Substrate Binding is Promoted by MEP50 and Regulated by Substrate PTMs

**Figure 6:**
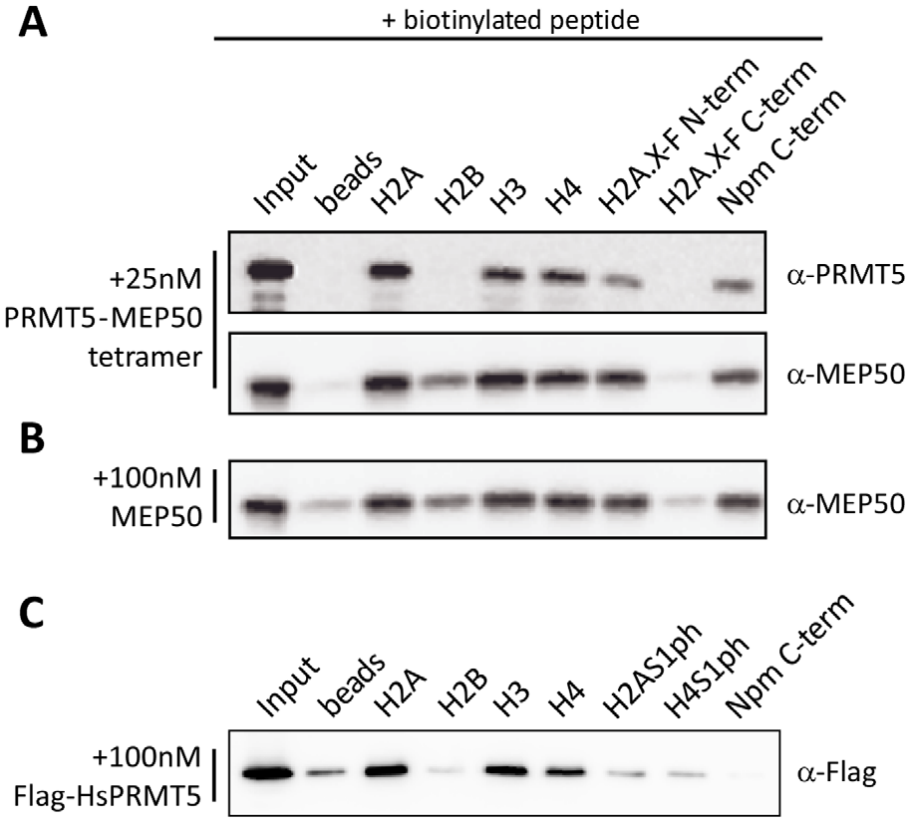
PRMT5 and MEP50 substrate binding. A. Biotinylated histone peptides [H2A, H2B, H3, H4, and H2A.X-F (all residues 1–20), and H2A.X-F (119–138)] and Npm (176–196) bound to streptavidin beads were incubated with 25 nM (tetramer) PRMT5-MEP50 complex or **B.** 100 nM MEP50. Captured protein was immunoblotted as indicated. “Beads” indicates no peptide. **C.** Biotinylated histone peptides [H2A, H2B, H3, H4, and H2A and H4 with phosphorylated S1 (S1ph)] and Npm (176–196) bound to streptavidin beads were incubated with 100 nM (monomer) Flag-HsPRMT5. Captured protein was immunoblotted.

To further test the hypothesis that MEP50 presents substrate to PRMT5, we probed PRMT5-MEP50 complex and MEP50 alone for peptide substrate interactions. Qualitative peptide-pulldown assays showed that PRMT5-MEP50 (Figure 6a) as well as MEP50 alone (Figure 6b) interacted with H2A/H2A.X and H4 N-terminal tail peptides, but not H2B N-terminal or H2A.X-F C-terminal peptides (Figure 6a). Intriguingly, the proteins interacted with histone H3 tail peptides even though the complex does not methylate H3 peptide in *in vitro* assays (Figure 4a).

Next, we incubated Flag-tagged *Hs*PRMT5 with histone peptides in qualitative pulldown assays to test if PRMT5 alone can bind to substrates (Figure 6c). Flag-*Hs*PRMT5 interacted with H2A, H3, and H4 as did the *Xenopus* complex. Its binding to the histone peptides was abrogated by S1ph. However, it was not enriched with the Nucleoplasmin C-terminal tail peptide.

### Substrate Binding to MEP50

**Figure 7:**
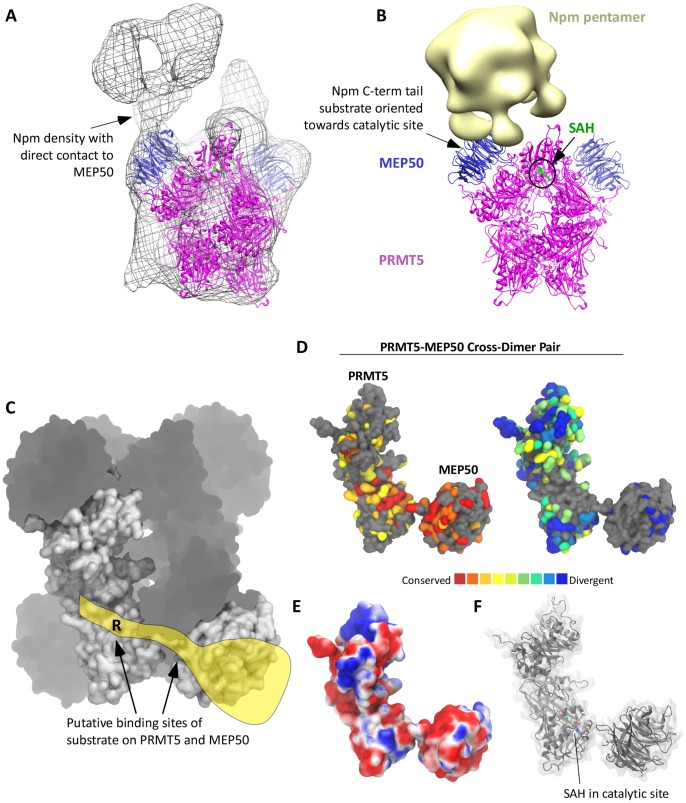
MEP50 serves as a substrate presenter for PRMT5 . **A.** Electron microscopy and reconstructed fitting of PRMT5-MEP50 (pink and blue) complexed with its substrate Nucleoplasmin. Electron density map shown in wire mesh. **B.** Electron microscopy and reconstructed fitting of PRMT5-MEP50 (pink and blue) complexed with its substrate Nucleoplasmin (Npm) modeled from density map EMD-1778 (gold). Npm is a stable homopentamer, with five C-terminal poorly structured “fingers” each containing the target sequence “…GRGRK…” (underlined R is methylated) [5], [35]. The position of the catalytic site is circled and noted by SAH in green. Electron density map is not shown. **C.** Surface and ghosted figure showing the PRMT5-MEP50 tetramer. A “cross-dimer” pair of PRMT5 and its corresponding dimer-bound MEP50 is shown in surface representation. The yellow illustrates a substrate interacting with a cross-dimer pair. The substrate arginine position is shown as “R”. **D.** The cross-dimer pair is shown in surface representation with evolutionarily conserved and divergent residues colored. Fully conserved residues are in red, substantially conserved residues are in orange and yellow. Highly divergent residues are in blue and green. Residues that are gray in both have insufficient data for conservation annotation. **E.** Electrostatic surface of the cross-dimer pair from a calculated Poisson-Boltzman analysis is shown, with red surfaces acidic and blue surfaces basic. **F.** The cross-dimer pair is illustrated in a cartoon model, with the catalytic site SAH illustrated. Sulfur position in yellow.

Our data presented so far support MEP50 binding substrate protein and stimulating PRMT5 activity. To further understand the role of MEP50, we aimed to locate substrate on PRMT5-MEP50 using electron microscopy (EM). We imaged *Xl*PRMT5-MEP50 in the absence or presence of Nucleoplasmin. Nucleoplasmin is a PRMT5-MEP50 substrate [5], is highly stable [33], [34], and had previously been imaged via EM [35]. Reconstructed PRMT5-MEP50 only contained two MEP50 molecules for the PRMT5 tetramer. Nevertheless, there was a pronounced increase in electron density centered on MEP50 in the class average projection and 3D reconstruction upon addition of Nucleoplasmin (Figure 7a,b and Figure S7). This density is clearly assigned to Nucleoplasmin in direct contact with MEP50. The substrate arginine in Npm (R187) is found on its disordered extreme C-terminal “fingers” [5].

## Discussion

We determined the crystal structure of the full-length *Xl*PRMT5-MEP50 complex and found a novel arrangement of PRMT5-MEP50 dimers that assembles to form a tetramer. Our data conclusively show that *Xl*PRMT5 forms a homotetramer in the crystalline state and in solution. The residues that make intra-tetramer contacts are 100% conserved between *Xenopus,* human and cattle PRMT5 and only have a single R to H change with mouse and rat PRMT5. There is no conservation in these residues between *Xenopus* PRMT5 and *Arabidopsis, Drosophila, C. elegans* PRMT5 or *S. cerevisiae* Hsl7. Superposition of the *C. elegans* structure and the *Xenopus* structure showed that the extensive loop found in the dimerization arm (residues 551 to 586 in *Ce*PRMT5 compared to residue 483 to 491 in *Xl*PRMT5) is incompatible with tetramerization as it would clash with the tetramer-paired PRMT5. These observations suggest that if non-vertebrate PRMT5 forms higher order assemblies it will be through a mechanism distinct from that responsible for the tetramer formation observed in our studies.

SAH–the product of the methyltransferase reaction–is substantially buried, implying that the SAM methyl donor is possibly accessible to substrate through a modest channel that connects the catalytic site with bulk solvent. Each active site is oriented in line with the cross-dimer paired MEP50. We showed that MEP50 is required for significant PRMT5 methyltransferase activity. High-density histone peptide arrays and solution assays demonstrated that the substrate charge-modulating post-translational modifications of lysine acetylation and serine phosphorylation stimulated or inhibited PRMT-MEP50 activity, respectively.

The function of the tetramer is unclear, and many residues will need to be mutated to disrupt the complex to test its function. The tetramer may serve to promote processive methylation of neighboring substrates (such as in a Nucleoplasmin:histone complex or on chromatin). PRMT5 has been observed interacting with many proteins in addition to MEP50. Therefore, the tetramer may act as a landing-pad for other proteins – such as pICln, RioK1 and CoPR5– that are known interactors and modulators of PRMT5 activity [21], [36], [37]. pICLn, a mammalian protein, associates with human PRMT5 and stimulates its methyltransferase activity towards Sm proteins [38]. However, increasing concentration of pICLn inhibited the already modest activity of PRMT5 towards histones in those studies [38], suggesting that pICLn may be involved in recognition of non-histone substrates.

All PRMTs published to date form dimers or higher order structures. A long-standing issue has been the functional and mechanistic basis for this dimerization. One possibility would be to enhance processive dimethylation by allowing two neighboring active sites to function together by successive turnover on a bound substrate. Our previous study showed that only increased concentration of PRMT5-MEP50 promoted dimethylation, consistent with a distributive catalysis mechanism [5]. PRMT5, in the absence of MEP50, only monomethylated substrate [28]. An alternative hypothesis is that dimerization is required for proper substrate recognition and binding. The results from this study favor the latter hypothesis. This is also consistent with the presence of divergent N-terminal domains in all PRMTs, implying that the N-terminus may be important for substrate recognition and binding. MEP50 facilitation of substrate binding and its location bound to the PRMT5 N-terminus support this idea.

PRMT5 typically coexists with MEP50 and the functional nature of this complex has long been questioned. MEP50 has been previously shown to interact with histone H2A [39]. Our studies, consistent with the recent finding by Antonysamy *et al*. [28] show that: 1) one PRMT5 is associated with one MEP50; 2) MEP50 binds on the distal side of PRMT5 from the active site; 3) MEP50 is required for significant PRMT5 methyltransferase activity; 4) MEP50 independently binds to the peptide and protein substrates; 5) substrate charge-modulating post-translational modifications of lysine acetylation and serine phosphorylation stimulated or inhibited PRMT-MEP50 activity, respectively. While human and *Xenopus* PRMT5 and MEP50 are highly identical, there is no obvious MEP50 ortholog in *C. elegans*. The low sequence identity (28.6% using MAFFT alignment) between *Xl*PRMT5 and *Ce*PRMT5 suggests that *Ce*PRMT5 may interact with substrate using a different mechanism.

Based upon this extensive array of structural and functional studies, we propose a “cross-dimer” substrate recognition model for PRMT5-MEP50 activity (Figure 7c). In our model MEP50 binds to the substrate distal of the target arginine and orients the unstructured substrate tail towards the catalytic site of the PRMT5 molecule that is not directly coupled to the substrate-bound MEP50. This hypothesis is supported by mapping conserved residues on PRMT5 and MEP50 (Figure 7d). The most conserved residues are highly enriched on the surfaces that we propose are involved in substrate interaction. Furthermore, the electrostatic surface features of this cross-dimer pair are appropriate for the recognition of positively-charged substrates (e.g. RGG or GRGK motifs) (Figure 7e). The position of the catalytic site in the cross-dimer pair model is illustrated in Figure 7f.

The invariant “double-E” loop is found in all arginine methyltransferases and is absolutely required for activity. This conservation may result from the need to productively position the ω and ω’ guanidino nitrogens for nucleophilic attack on the S-methyl group of SAM [25]. In the *Xenopus* PRMT5-MEP50 structure we observed that these invariant glutamic acid residues (Glu431 and Glu440) are hydrogen bonded to SAH, in contrast to the *C. elegans* and human structures (Figure S8). This may reflect an alternative role for these residues in ordering SAM for catalysis, or it may indicate that a conformational change occurs post-catalysis leading to SAH forming new bonds. The unambiguous density for the adenosyl moiety in the *Xenopus* structure presented here conclusively demonstrates that the hydrogen bonds between the conserved glutamates and SAH are present. Furthermore, the SAH pose here is incompatible with the substrate arginine entry shown for human PRMT5. A co-crystal structure with the arginine-containing substrate, preferably in a catalytically trapped state, will be necessary to parse the mechanism of action for the *Xenopus* PRMT5.

While this manuscript was under review, the structure of human PRMT5-MEP50 was determined in the presence of a short histone H4 peptide (PDB:4GQB) and a SAM analog rather than SAH [28]. In the *Xenopus* structure, the aminoethanoic acid pose of SAH would impair arginine entry through the pocket described in the human structure (Figure 1e and *not shown*). However, our analysis shows an alternative channel, exposed to bulk solvent, which would permit arginine guanidinium entry in line with the SAH sulfur and the two catalytically important glutamic acid residues. Intriguingly, these invariant glutamic acid residues (Glu431 and Glu440) in the “double-E” loop are hydrogen bonded to aminoethanoic acid of homocysteine in our model. Our model may provide another mode for PRMT5 substrate interaction and would allow peptide interact with the dimer-paired MEP50 (Figure 1d, circled pocket 1). If the entry found in the human structure is utilized by the *Xenopus* PRMT5, the SAH pose we presented here may represent a post-catalysis state.

We observed inhibition of PRMT5 activity when the substrate contained Ser1 phosphorylation. We suggest that this charge-shifting PTM may displace the unstructured substrate tail and displace the arginine, two amino acids away, from the active site. Interestingly, this modification is enriched in mitotic and S-phase cells [40] and early embryos [41]. We also observed increased PRMT5 activity towards acetylated histones, both from HeLa cells treated with butyrate and on the peptide array. The substrate charge-shifting acetylation likely serves to modulate enzyme-substrate interaction or turnover, leading to increased activity, consistent with previously published results for PRMT5 [42]. Many of these acetylation marks are enriched on histones prior to chromatin incorporation [41]. This suggests a mechanism of constraining PRMT5 activity to undeposited histones. This conclusion is consistent with the absence of PRMT5-MEP50 activity on nucleosomes, as the PRMT5-MEP50 surface negative charge might be repelled by negatively-charged DNA on the nucleosome surface. Further study, including kinetic measures of PRMT5-MEP50 activity with various substrates, will be required to decipher this mechanism.

## Materials and Methods

Chemicals and reagents were obtained from Sigma (St. Louis, MO) or Fisher Scientific (Pittsburgh, PA). Sf9 and High Five cells were grown in Sf-900 III with L-glutamine and Express Five Medium (Invitrogen, Carlsbad, CA), respectively, with penicillin and streptomycin. Recombinant Flag-*Hs*PRMT5 enzyme was purchased from SinoBiologicals (Beijing, China). HeLa octamers and mononucleosomes were purified as described after growth in the presence or absence of 40 mM Na Butyrate for 16 hours [43]. Baculovirus containing genes for *Xenopus* PRMT5 and MEP50 were produced as described [5].

### Purification of Recombinant XlPRMT5-MEP50

PRMT5 and MEP50 proteins were co-expressed in Hi5 cells by infecting 500 ml of 2×10^6^ cells/ml culture with baculovirus while shaking at 27°C for 60 hours [5]. Cells were pelleted and suspended in cold lysis buffer (50 mM HEPES pH 7.4, 10% glycerol, 250 mM NaCl, 1 mM PMSF and 1 mM β-mercaptoethanol) and lysed by an EmulsiFlex-C5 homogenizer (Avestin, Ottawa, Canada) followed by 12000×g centrifugation for 60 min. The protein was applied to a Ni-NTA column (Qiagen) and eluted with 20 mM ADA pH 6.5, 10% glycerol, 250 mM NaCl, 5 mM DTT and 250 mM imidazole. It was immediately diluted with two volumes of the elution buffer without imidazole, spin-concentrated and applied to a Superdex 200 16/60 column. Peak complex fractions were pooled and spin-concentrated.

### Production of Selenomethionine-substituted XlPRMT5-MEP50

Hi5 cells were adapted to ESF-921 medium without methionine (Expression Systems). *Xl*PRMT5-MEP50 protein complexes were co-expressed in Hi5 cells with baculovirus in ESF-921 medium without methionine while shaking at 27°C. 100 mg/L of D,L-Selenomethionine was added to the medium 20 h post-infection and an additional 50 mg/L of D,L-selenomethionine was added 44 h post-infection. The cells were harvested 71 hour post-infection and purified.

### Crystallization, Data Collection, and Structure Determination of XlPRMT5-MEP50 Complexes

*Xl*PRMT5-MEP50-SAH complexes were prepared by incubating ∼8–10 mg/ml *Xl*PRMT5-MEP50 in 400–500 mM NaCl, 10% glycerol, 5 mM DTT and 20 mM ADA pH 6.5 on ice with 1 mM SAH. The complex was co-crystallized in 30%–40% MPD and 100 mM Bis-Tris pH 5.5 at 18°C using hanging drop or sitting drop vapor diffusion methods. Crystals were directly flash frozen in liquid nitrogen before data collection. X-ray diffraction data were collected at the X25 beamline of Brookhaven National Laboratory on a Pilatus 6 M detector at 100° K. The data were processed with the HKL2000 program suite [44] and is summarized in Table 1. A Ramanchran plot shows that only 0.4% of residues are in the disallowed region.

Crystals of selenomethionine substituted PRMT5-MEP50 had sufficient incorporation to allow identification of a subset of the labeled sites with the HKL2MAP suite of programs [45]. Initial phases were calculated using MLPHARE [46] based on 13 of the final 27 modeled selenium sites. Improved phases, calculated using DM [47], allowed the visual identification of secondary structural features in the resulting electron density map. A model of *Xl*PRMT5 was calculated based on the structure of *Ce*PRMT5 (PDB entry 3UA3 [26]) using Phyre2 [48]. Two copies of the C-terminal portion of the model were placed in the electron density map using a combination of rotation function and phased translation function as implemented in the program Molrep [49]. The two fold-rotational non-crystallographic symmetry (NCS) operator was calculated using O [50] and improved using the USF Rave package of programs [51]. The C-terminal PRMT5 model (two protomers) was refined using rigid-body and torsion angle refinement protocols with Refmac5 [52] and the resulting phases were improved by taking advantage of NCS using DM. At this stage, additional density features attributable to the N-terminal fragment of PRMT5 and MEP50 became discernible in the 2mF_o_-DF_c_ maps. Two copies of the N-terminal fragment of PRMT5 were placed in the averaged electron density map, following a similar method as for the C-terminal fragments, and were refined with Refmac5. The N- and C-terminal fragments thus placed were observed to follow nearly identical NCS operators. A model of MEP50, based on PDB entry 2H9L, was placed in the refined 2mF_o_-DF_c_ electron density as described above using Molrep [49]. Only one copy of MEP50 could be reliably positioned using automated methods, presumably due to the more distant sequence relationship between MEP50 and the model. The second MEP50 protomer was placed based on the existing NCS relationship of the two PRMT5 molecules and agreement between the second MEP50 molecule and local electron density features. An anomalous difference Fourier synthesis using intermediate refined phases revealed strong peaks in the vicinity of all PRMT5 methionine-Se positions. The position of the single ordered Met in MEP50 was also revealed in this analysis and required the MEP50 models to be rotated by one blade of the beta propeller to place the methionine in proximity to the anomalous peak.

Models without SAH were iteratively rebuilt in COOT and refined in Phenix [53], [54]. Manual SAH building was initiated only after the R_free_ decreased below 35% and was guided by clear ligand density in F_o_–F_c_ electron density maps contoured at 3σ. Data processing and refinement statistics are summarized in Table 1.

### Structure Analysis and Visualization

Poisson-Boltzman calculations were performed using the PDB2PQR web server [55] and APBS [56]. Electrostatic potential maps and all figures were visualized using VMD v1.9.1[57]. Surface conservation was mapped with ConSurf [58].

### Analytical Ultracentrifugation

Sedimentation equilibrium and velocity experiments with the PRMT5-MEP50 complex protein were performed with a Beckman XL-I analytical ultracentrifuge using the absorption optics set to 280 nm. The buffer density and the partial specific volume of the complex were calculated using the Sednterp software (http://www.rasmb.bbri.org). For the equilibrium experiments, complex at three concentrations (0.23, 0.77 & 0.9 µM tetramer) in 20 mM ADA, 250 mM NaCl, 10% (v/v) glycerol and 5 mM DTT at pH 6.5 were loaded into the six channel cells. The samples were sequentially equilibrated at rotor speeds of 5,000 and 9,000 rpm for 24 hr each at 20°C in a Ti-60 rotor. The sedimentation equilibrium data were analyzed using version 1.1.44 of HeteroAnalysis [59]. The sedimentation velocity analysis was conducted in double sector cells at 0.97 µM at 30,000 rpm and 20°C in a Ti-60 rotor in the same buffer as the equilibrium experiments. The data were analyzed using version 2.3.4 of DCDT+ [60].

### Small Angle X-ray Scattering

SAXS was performed using Bio-SAXS beam line BL4-2 at Stanford Synchrotron Radiation Lightsource (SSRL) [61]. All data were collected on a Rayonix MX225-HE CCD detector (Rayonix, Evanston, IL) with a 1.7 m sample-to-detector distance and a beam energy of 11 keV (wavelength, λ = 1.127 Å) was used. The momentum transfer (scattering vector) q was defined as q = 4Πsin (θ)/λ, where 2θ is the scattering angle. The q scale was calibrated by silver behenate powder diffraction [62] and all data were collected up to a maximum q of 0.53 Å^−1^. The details of the FPLC-SAXS experiment at BL4-2 were described previously [63].

The data acquisition program Blu-ICE [64], [65] was employed for data collection and the data processing program SasTool (http://ssrl.slac.stanford.edu/~saxs/analysis/sastool.htm) was used for scaling, azimuthal integration and averaging of individual scattering images after inspection for any variations potentially caused by radiation damage. The first 100 images were scaled and averaged to create a buffer-scattering profile, and this was then subtracted from each of the subsequent images to produce the final scattering curve for each exposure. The 9 scattering profiles over an elution peak were averaged and then used for curve fitting with crystal structure using the program CRYSOL [32]. Pairwise distribution functions P(r) were calculated up to q = 0.3 using the program GNOM [66].

### Size-Exclusion Chromatography – Multi-Angle Light Scattering (SEC-MALS)

15 µg of PRMT5 and MEP50 in 20 mM ADA pH 6.5, 250 mM NaCl, 10% glycerol, and 5 mM DTT was subjected to size exclusion chromatography using a WTC030N5 (Wyatt Technology Corporation) column coupled to a Shimadzu HPLC system. Light scattering measurements were performed downstream, using a miniDawn TREOS instrument connected to the column output, followed by Optilab rEX refractive index analysis (Wyatt Technology Corporation). Control experiments were carried out with BSA diluted in the same buffer as the sample buffer. Data from these experiments was collected and interpreted using Astra software (version 6.0.3.16).

### Peptide Pulldown

5 µg biotinylated peptides were incubated with 20 µl magnetic streptavidin-coupled beads (New England Biolabs, 50% slurry) in PBS (137 mM NaCl, 2.7 mM KCl, 4.3 mM Na_2_HPO_4_, 1.47 mM KH_2_PO_4_, 1 mM PMSF, pH 7.4) for 3 hours at room temperature. After washing the beads three times with 500 µl PBS/0.1% Tween, they were incubated with 100 nM recombinant protein in PBS over night at 4°C. The next day the beads were washed four times with 500 µl Buffer D (20 mM Hepes pH 7.9, 20% glycerol, 0.2 mM EDTA, 0.1% Triton X-100, 300 mM KCl, 1 mM PMSF, protease inhibitors) and once with 500 µl Lower Hepes (4 mM Hepes, pH 7.9, 10 mM NaCl, 1 mM PMSF, protease inhibitors). Finally the beads were resuspended in 50 µl Tris (0.1 M, pH 8.0) and 10 µl 6x loading dye and boiled at 95°C. The protein samples were separated via SDS PAGE together with an input control (5%) followed by western blot analysis.

### Depletion Assay

Protein-A-sepharose beads (GE Healthcare) were coupled to anti-MEP50 antibodies or pre-immune serum (as control) for 1.5 hours in HNTG buffer (20 mM Hepes pH 8.0, 50 mM KCl, 0.1% Triton, 10% glycerol, 0.1 M EDTA and 1 mM PMSF). After washing the beads, 50 µl egg extract (HSS) were incubated with the first half of the antibody-coupled beads for one hour, followed by a second round of depletion with the other half. The MEP50-depleted egg extract was used for further analysis via western blot and methyltransferase activity assay.

### HeLa Mononucleosome Preparation

Nuclei were isolated from HeLa cells and MNase digested in TM2 buffer [67]. Mononucleosomes were then obtained using salt extraction in 250 mM NaCl-buffer (250 mM NaCl, 10 mM Tris pH 7.4, 2 mM MgCl_2_, 2 mM EGTA, 0.1% Triton X-100, 0.5 mM PMSF) for one hour at 4°C.

### Histone Methyltransferase Assay

Histone 1–20 peptides (Anaspec, Fremont, CA) or Npm peptides (amino acids 176–196, synthesized at the Rockefeller University Peptide Synthesis Service) at 3.3 µM final concentration, or 0.5 µg full-length histones or histone complexes, were incubated with 50–220 nM recombinant PRMT5-MEP50 and 0.5 µM ^3^H-SAM in 15 µl reaction buffer (20 mM Tris pH 8.0, 10 mM DTT, protease inhibitors) for 20 min at 30°C. The reconstituted Flag-*Hs*PRMT5-*Xl*MEP50 was pre-incubated in equimolar amounts for 15 min at room temperature prior to the reaction. The reaction mix was spotted on P81 filter paper, washed with sodium carbonate buffer (0.1 M, pH 8.5), air-dried and analyzed via scintillation counter (Wallac Winspectral 1414 LSC).

### Flag-pulldown

20 µl Anti-Flag M2 antibody-coupled agarose beads were incubated with equimolar amounts (50 nM) of *Xenopus* MEP50 and Flag-*Hs*PRMT5 in TBS at 4°C. As a negative control, MEP50 was incubated under the same conditions without PRMT5. After 2 hours, the suspension was transferred to a Mini-spin column and centrifuged (30 sec, 500 g). After washing, the proteins were eluted from the beads and analyzed via western blot (α-PRMT5 and α-MEP50 antibodies).

### Histone Code Peptide Microarrays

A library of 20-mer peptides spanning the sequences of histones H2A (P0C0S8), H2B (P62807), H3 (P68431) and H4 (P62805) was generated including single known modifications and in various combinations (sequences available at www.jpt.com). Peptides synthesized using spot synthesis [68] were chemoselectively immobilized onto functionalized glass slides as described earlier [69]. For activity assays 10 µg/mL PRMT5-MEP50 complex was incubated in KCl/HEPES buffer (100 mM KCl (pH 7.5), 20 mM HEPES, 1 mM EDTA, 0.1 mM DTT, and 10% glycerol) in presence or absence of 1.5 mM S-adenosylmethionine (New England Biolabs, Ipswich, MA). Reactions were carried out on peptide microarrays at 30°C for 12 hours in a humidity chamber. Detection of methyltransferase activity was performed in a Tecan HS4800 microarray processing station [70]. The microarrays were incubated with anti-H4R3me2s rabbit polyclonal antibody (Millipore, #07–947) followed by washing and incubation with fluorescently labeled secondary antibody (DL649-anti-rabbit IgG; Pierce, #35565). Activity was represented as the ratio of the R3me2s antibody fluorescence signal in the presence (+SAM) and absence (-SAM) of the methyl donor in the reaction. Each microarray was scanned using GenePix Autoloader 4200AL (Molecular Devices, Pixel size: 10 µm). Signal intensity was evaluated using GenepixPro software (Molecular Devices). Further evaluation and representation of results was performed using the R statistical programming system (Version 2.11.1, www.r-project.org).

### Electron Microscopy and Image Analysis

PRMT5-MEP50 alone or in complex with Npm (at a molar ratio of 1∶2) were applied to carbon-coated copper grids and negatively stained for ∼30 seconds with 2% uranyl acetate. Micrographs viewed on a JEOL JEM-2100F electron microscope were recorded on a TVIPS TemCam-415 CCD camera with a pixel size of 1.67A at specimen space. Images of individual of PRMT5-MEP50 were boxed out and classified into homogeneous classes using multivariant statistical analysis and the selected class averages were used to build initial three-dimensional density model. An alternative density model was computed from those images with strong C2 symmetry and the images with strong mirror symmetry. Refinement was carried out by confronting data images with calculated projections from the density models using methods implemented as previously described [71], [72]. C1 symmetry was superimposed in the refinement as well as the reconstruction of PRMT5-MEP50-Npm complex, while C2 symmetry was applied in the processing of PRMT5-MEP50 alone. The reconstructions were converged to a stable and consistent density map in both cases, although the initial models were built with two independent methods. In total, 2294 and 1250 images were retained in the final reconstruction of PRMT5-MEP50-Npm complex and PRMT5-MEP50, respectively. Model fitting was done manually with the crystal structure of PRMT-MEP50 with UCSF chimera (http://www.cgl.ucsf.edu/chimera) and then refined with automatic fitting package Situs [73]. The Npm density map (EMD-1778) [35] was obtained from EMDB (http://www.emdatabank.org).

## Supporting Information

Figure S1 **PRMT5 and MEP50 domain organization and structural homology.** **A.** *Xl*PRMT5 domain organization, with the top plot highlighting the major subdomains, including the TIM barrel, Rossmann fold (location of nucleotide binding) and a β-barrel fold. The interacting residues determined in the structure are shown on top. The lower half shows the discrete structural domains and the flexible connector loop between the N- and C-terminal units. The inset boxes show the *Xenopus* N- and C-terminal domains overlaid over the *Ce*PRMT5 structure (3UA3). **B.** *Xl*MEP50 organization, with the top plot indicating the location of the “cross-dimer insertion loop” (yellow) and the residues that form explicit contacts with PRMT5 (green). The lower part shows the structure and highlights the insertion loop in yellow. The inset shows MEP50 overlaid with WDR5 (2H9M). The location of residues missing from the MEP50 structure is indicated. **C.** An electrostatic potential map of *Xl*PRMT5-MEP50 is plotted from 3 different perspectives, as indicated, with red surfaces acidic and blue surfaces basic.
(PDF)

Figure S2 **PRMT5 conservation across evolution and alignment.** **A.** PRMT5 amino acid identity was calculated using the MAFFT alignment in Geneious v5.5. **B.** A PRMT5 multiple sequence alignment (without *S. cerevisiae* Hsl7) is shown with conserved residues positions highlighted in black and divergent residues in white. Locations of interaction domains determined in the structure are highlighted above the plot.
(PDF)

Figure S3 **The SAH-omitted electron density map near the SAH binding site.** The SAH molecule and some surrounding residues are drawn in gray and yellow color, respectively. The mF_o_-F_c_ map (difference map) at 3 σ is shown in green and the 2F_o_-F_c_ map at 1.5 σ is shown in blue.
(PDF)

Figure S4 **PRMT5 interacting residues with SAH, dimer interface, tetramer interface, and with MEP50. A.** Residues that we identified in the structure interacting with SAH are shown, with their corresponding hydrogen bonded atom in SAH and the distance in angstroms. *indicates residues that may also be involved in catalysis. **B.** Ligplot representation of the hydrogen bonding and neighboring residues around SAH in the structure. Inset: SAH pose from 3UA3. **C.** PRMT5 dimer interface residues, split into salt bridges in the dimerization arm and salt bridges and hydrogen bonds in the N- and C- terminal domains. **D.** PRMT5 tetramer interface residues, split into salt bridges and hydrogen bonds, with distances listed in angstroms. **E.** PRMT5 and MEP50 interacting residues are shown, with salt bridges, cation-Π interactions and hydrogen bonds illustrated. **F.** Cartoon representation of the PRMT5 152–178 loop and its interactions with MEP50.
(PDF)

Figure S5 **R3me2s antibody response on peptide array.** A high-density peptide array was probed with R3me2s antibody (Millipore #07–947) to measure the baseline signal to determine if neighboring PTMs modulate the response. Peptides are listed in text on the left. In the middle, black boxes represent the presence of a particular modification on a peptide. The histogram on the right shows the relative antibody signal. Pink bar shows the signal on the H4(1–20)R3me2s peptide. Green boxes show the presence of R3me1 or R3me2s.
(PDF)

Figure S6 **Recombinant human PRMT5 is not complexed with MEP50.** **A.** Recombinant human PRMT5 produced in 293 cells was immunoblotted for PRMT5 (Coomassie stain on left, immunoblot on right). **B.** Recombinant GST-tagged human MEP50 and human PRMT5 were immunoblotted with an antibody specific for human MEP50 (membrane stain on left, immunoblot on right).
(PDF)

Figure S7 **Electron microscopy and reconstruction.** **A.** Recombinant class average 2D projections of PRMT5-MEP50. **B.** PRMT5-MEP50 incubated with recombinant Nucleoplasmin class average 2D projections. The additional density from Nucleoplasmin was observed centered on MEP50 (yellow arrows). **C.** 3D electron microscopy reconstruction of PRMT5-MEP50 complexed with Nucleoplasmin. PRMT5-MEP50 and Nucleoplasmin molecules from the structure were placed in the density map. The density map is shown wire mesh.
(PDF)

Figure S8 **SAH Pose Comparison. A.** SAH in *Xenopus* PRMT5 (PDB:4G56). H-bonds are indicated as dashed yellow lines. The sulfur atom is shown in yellow. **B.** SAH in human PRMT5 (PDB:4GQB), H4R3 in green, PRMT5 in pink. **C.** SAH of *Xenopus* and human PRMT5 overlaid. The aminoethanoic acid in the *Xenopus* structure clashes with the H4R3 guanidinium position in the human structure. **D.** SAH of rat PRMT1 (Cyan, PDB:1ORI), human PRMT3 (Yellow, PDB:2FYT), mouse PRMT4 (Gray, PDB:2V74), *C. elegans* PRMT5 (Pink, PDB:3UA3), and human PRMT5 (Pink, 4GQB).
(PDF)
