# Correction: Structure of the Arginine Methyltransferase PRMT5-MEP50 Reveals a Mechanism for Substrate Specificity

**DOI:** 10.1371/annotation/e6b5348e-9052-4a3b-8f06-90d01dc88fc2

**Published:** 2013-08-20

**Authors:** Meng-Chiao Ho, Carola Wilczek, Jeffrey B. Bonanno, Li Xing, Janina Seznec, Tsutomu Matsui, Lester G. Carter, Takashi Onikubo, P. Rajesh Kumar, Man K. Chan, Michael Brenowitz, R. Holland Cheng, Ulf Reimer, Steven C. Almo, David Shechter

There was an error in the third from last statement in the Funding section.

The correct sentence is "This research was partially supported by grants from Academia Sinica, Taiwan, and from the National Science Council, Taiwan (NSC101-2311-B-001-002) to MHC, the NIH (U54GM094662 to SCA and AI095382 to RHC) and the Albert Einstein Cancer Center (CA013330)." 

