# Supplementary material for: Structure of the Arginine Methyltransferase PRMT5-MEP50 Reveals a Mechanism for Substrate Specificity
Source: PLoS One. 2013 Feb 25;8(2):e57008. doi: 10.1371/journal.pone.0057008 (PMC3581573; doi:10.1371/journal.pone.0057008)
Supplement: Figure S1 — PRMT5 and MEP50 domain organization and structural homology. A. XlPRMT5 domain organization, with the top plot highlighting the major subdomains, including the TIM barrel, Rossmann fold (location of nucleotide binding) and a β-barrel fold. The interacting residues determined in the structure are shown on top. The lower half shows the discrete structural domains and the flexible connector loop between the N- and C-terminal units. The inset boxes show the Xenopus N- and C-terminal domains overlaid over the CePRMT5 structure (3UA3). B. XlMEP50 organization, with the top plot indicating the location of the “cross-dimer insertion loop” (yellow) and the residues that form explicit contacts with PRMT5 (green). The lower part shows the structure and highlights the insertion loop in yellow. The inset shows MEP50 overlaid with WDR5 (2H9M). The location of residues missing from the MEP50 structure is indicated. C. An electrostatic potential map of XlPRMT5-MEP50 is plotted from 3 different perspectives, as indicated, with red surfaces acidic and blue surfaces basic. (PDF) [file pone.0057008.s001.pdf]

doi: 10.1371/journal.pone.0057008

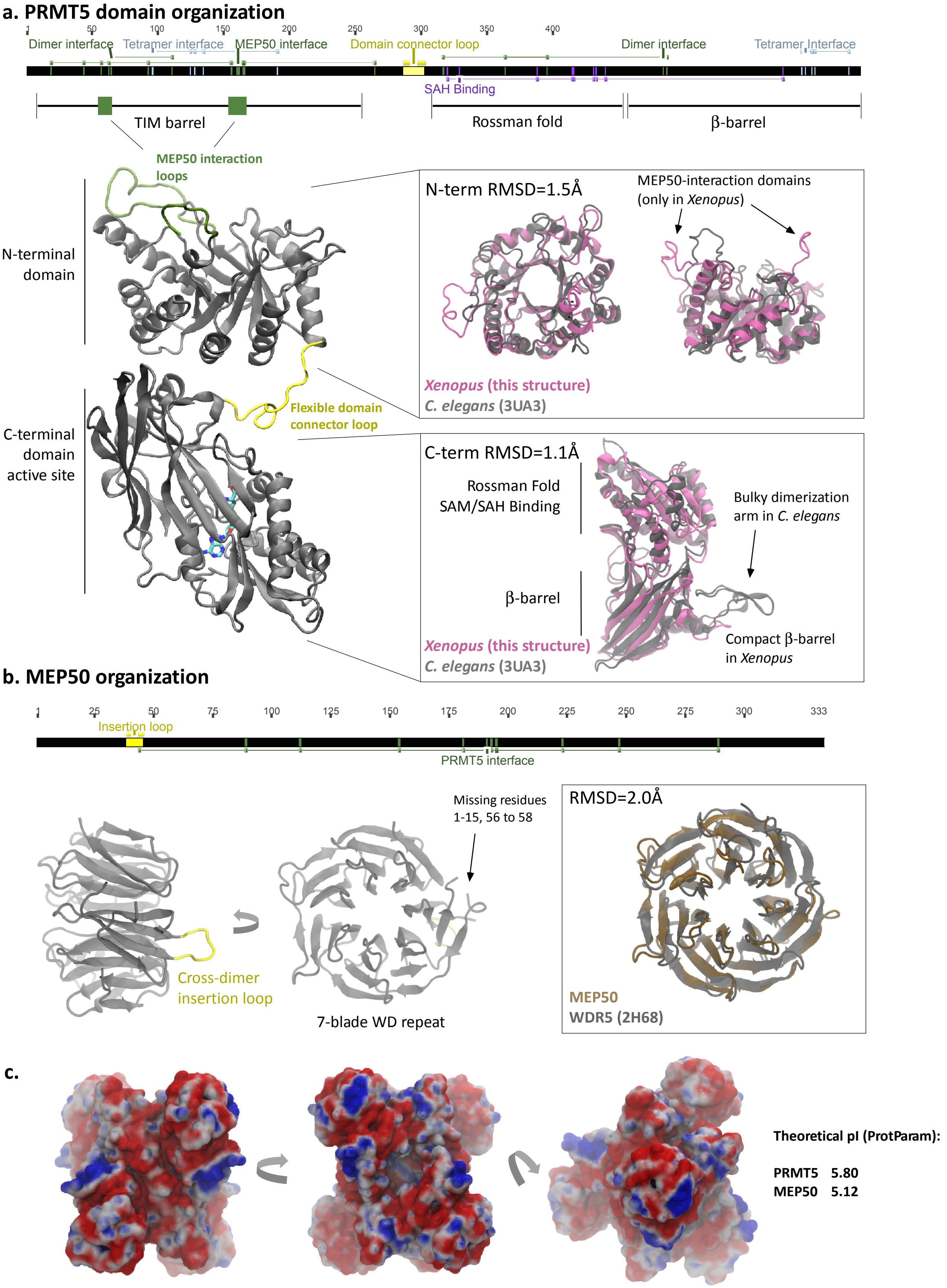

Supplemental Figure S1. PRMT5 and MEP50 domain organization and structural homology- A. XlPRMT5 domain organization, with the top plot highlighting the major subdomains, including the TIM barrel, Rossman fold (location of nucleotide binding) and a β-barrel fold. The interacting residues determined in the structure are shown on top. The lower half shows the discrete structural domains and the flexible connector loop between the N- and C-terminal units. The inset boxes show the Xenopus N- and C-terminal domains overlaid over the CePRMT5 structure (3UA3). B. XlMEP50 organization, with the top plot indicating the location of the "cross-dimer insertion loop" (yellow) and the residues that form explicit contacts with PRMT5 (green). The lower part shows the structure and highlights the insertion loop in yellow. The inset shows MEP50 overlaid with WDR5 (2H9M). The location of residues missing from the MEP50 structure is indicated. C. An electrostatic potential map of XlPRMT5-MEP50 is plotted from 3 different perspectives, as indicated, with red surfaces acidic and blue surfaces basic.
