# Supplementary material for: Structure of the Arginine Methyltransferase PRMT5-MEP50 Reveals a Mechanism for Substrate Specificity
Source: PLoS One. 2013 Feb 25;8(2):e57008. doi: 10.1371/journal.pone.0057008 (PMC3581573; doi:10.1371/journal.pone.0057008)
Supplement: Figure S2 — PRMT5 conservation across evolution and alignment. A. PRMT5 amino acid identity was calculated using the MAFFT alignment in Geneious v5.5. B. A PRMT5 multiple sequence alignment (without S. cerevisiae Hsl7) is shown with conserved residues positions highlighted in black and divergent residues in white. Locations of interaction domains determined in the structure are highlighted above the plot. (PDF) [file pone.0057008.s002.pdf]

doi: 10.1371/journal.pone.0057008

a. PRMT5 Percent identity (MAFFT alignment):

	Xenopus	Human	Zebrafish	Arabidopsis	Drosophila	C. elegans	S. cerevisiae
Xenopus PRMT5		83.4%	77.3%	43.8%	36.8%	28.6%	21.8%
Human PRMT5	83.4%		78.5%	44.4%	37.8%	29.2%	21.1%
Zebrafish PRMT5	77.3%	78.5%		44.4%	37.5%	29.9%	21.0%
Arabidopsis PRMT5	43.8%	44.4%	44.4%		33.6%	28.6%	21.2%
Drosophila PRMT5	36.8%	37.8%	37.5%	33.6%		26.7%	17.2%
C. elegans PRMT5	28.6%	29.2%	29.9%	28.6%	26.7%		17.1%
Sc Hsl7 (cerevisiae PRMT5)	21.8%	21.1%	21.0%	21.2%	17.2%	17.1%	

b. PRMT5 Multiple Sequence Alignment

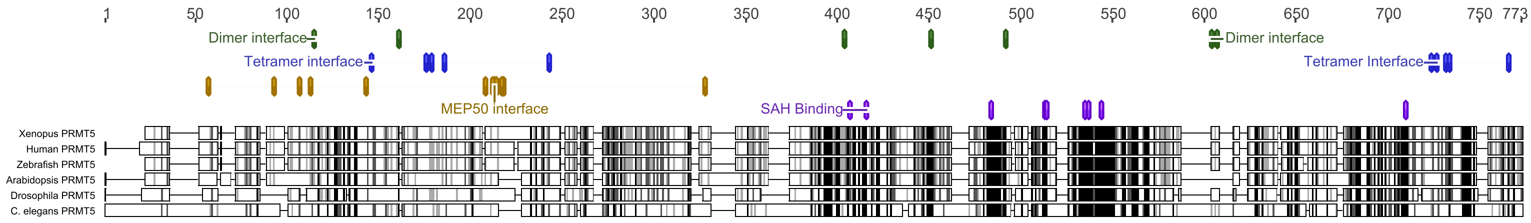

Supplemental Figure S2. PRMT5 conservation across evolution and alignment- A. PRMT5 amino acid identity was calculated using the MAFFT alignment in Geneious v5.5. B. A PRMT5 multiple sequence alignment (without *S. cerevisiae* Hsl7) is shown with conserved residues positions highlighted in black and divergent residues in white. Locations of interaction domains determined in the structure are highlighted above the plot.
