# Supplementary material for: Structure of the Arginine Methyltransferase PRMT5-MEP50 Reveals a Mechanism for Substrate Specificity
Source: PLoS One. 2013 Feb 25;8(2):e57008. doi: 10.1371/journal.pone.0057008 (PMC3581573; doi:10.1371/journal.pone.0057008)
Supplement: Figure S3 — The SAH-omitted electron density map near the SAH binding site. The SAH molecule and some surrounding residues are drawn in gray and yellow color, respectively. The mFo-Fc map (difference map) at 3 σ is shown in green and the 2Fo-Fc map at 1.5 σ is shown in blue. (PDF) [file pone.0057008.s003.pdf]

doi: 10.1371/journal.pone.0057008

SAH-omitted electron density map

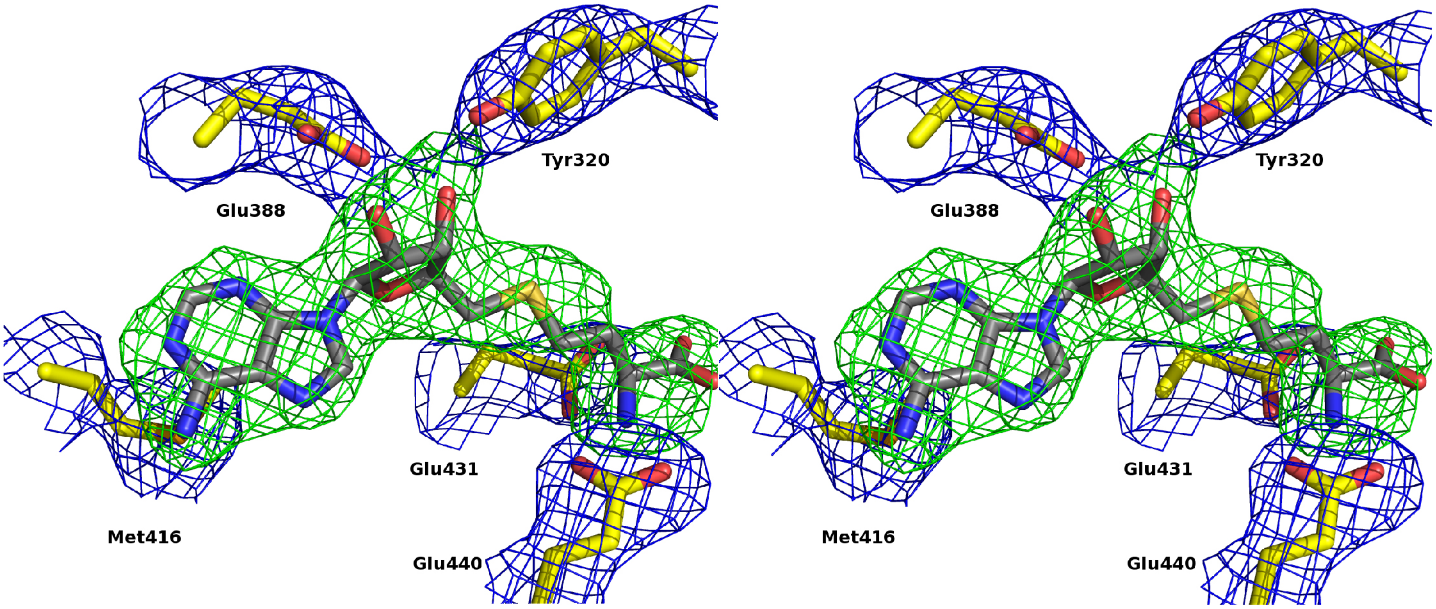

mF_o-DF_c Difference Map at 3σ

$2mF_o$-DF_c Map at 1.5σ

Supplemental Figure S3. The SAH-omitted electron density map near the SAH binding site- The SAH molecule and some surrounding residues are drawn in gray and yellow color, respectively. The mF_o-F_c map (difference map) at 3 σ is shown in green and the $2F_o$-F_c map at 1.5 σ is shown in blue.
