# Supplementary material for: Structure of the Arginine Methyltransferase PRMT5-MEP50 Reveals a Mechanism for Substrate Specificity
Source: PLoS One. 2013 Feb 25;8(2):e57008. doi: 10.1371/journal.pone.0057008 (PMC3581573; doi:10.1371/journal.pone.0057008)
Supplement: Figure S4 — PRMT5 interacting residues with SAH, dimer interface, tetramer interface, and with MEP50. A. Residues that we identified in the structure interacting with SAH are shown, with their corresponding hydrogen bonded atom in SAH and the distance in angstroms. *indicates residues that may also be involved in catalysis. B. Ligplot representation of the hydrogen bonding and neighboring residues around SAH in the structure. Inset: SAH pose from 3UA3. C. PRMT5 dimer interface residues, split into salt bridges in the dimerization arm and salt bridges and hydrogen bonds in the N- and C- terminal domains. D. PRMT5 tetramer interface residues, split into salt bridges and hydrogen bonds, with distances listed in angstroms. E. PRMT5 and MEP50 interacting residues are shown, with salt bridges, cation-Π interactions and hydrogen bonds illustrated. F. Cartoon representation of the PRMT5 152–178 loop and its interactions with MEP50. (PDF) [file pone.0057008.s004.pdf]

Ho, Wilczek, et al. 2013. Structure of the Arginine Methyltransferase PRMT5-MEP50 Reveals a Mechanism for Substrate Specificity
doi: 10.1371/journal.pone.0057008

a. PRMT5 Interaction with SAH

Hydrogen Bonds (*Xenopus* PRMT5 / this structure)

SAH	Distance (Å)	PRMT5
N6 of adenosine	2.5	Asp415 side chain
N1 of adenosine	3.1	Met416 amine
3'-hydroxyl of adenosine	2.7	Tyr320 side chain
amino of homocysteine	2.7	Glu440 side chain *
amino of homocysteine	2.6	Leu433 carbonyl
carboxyl of homocysteine	3.1	Lys329 side chain
carboxyl of homocysteine	2.9	Glu431 side chain *
carboxyl of homocysteine	3.2	Trp575 side chain
2'-hydroxyl of adenosine	2.9	Glu388 side chain

Hydrogen Bonds (*C. elegans* PRMT5 / 3UA3)

SAH	Distance (Å)	PRMT5
N6 of adenosine		Asp451 side chain
N1 of adenosine		Met478 amine
2'-hydroxyl of adenosine		Glu450 side chain
3'-hydroxyl of adenosine		Tyr376 side chain
amino of homocysteine		Glu499 side chain
carboxyl of homocysteine		Tyr386 side chain

b. LigPlot view of SAH interactions

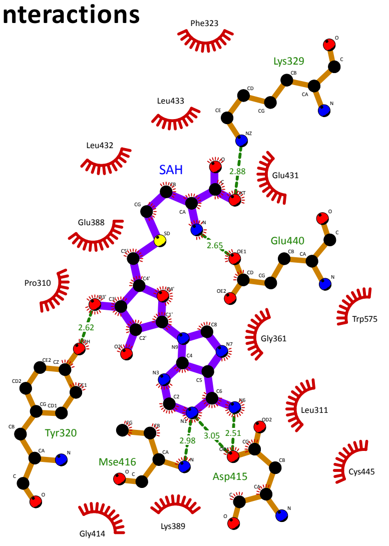

c. PRMT5 Dimer Interface

Dimerization Arm (Interaction region 1)

Salt Bridges

PRMT5 A	Distance (Å)	PRMT5 I
Arg484	2.6	Asp487
Asp487	2.6	Arg484

Head-to-tail / N-to-C (Interaction region 2)

Salt Bridges

PRMT5 A	Distance (Å)	PRMT5 C
Arg364	3.1	Asp65
Asp65	3.1	Arg364

Hydrogen Bonds

PRMT5 A	Distance (Å)	PRMT5 I
Thr396(OG1)	2.5	Asp65 (OD1)
Ser317 (N)	2.9	Tyr111 (O)
Asp 65 (OD1)	2.5	Thr396 (OG1)
Tyr 111 (O)	2.9	Ser317 (N)

d. PRMT5 Tetramer Interface

Salt Bridges

PRMT5 A	Distance (Å)	PRMT5 C
Asp 125	2.3	Arg597
Asp592	2.6	Arg589
Arg597	2.5	Asp125
Lys96	2.6	Asp527
Arg589	2.7	Asp592

Lys96(C) to Asp527(A) is missing. Lys96 side chain Nz is disordered.

Hydrogen Bonds

PRMT5 A	Distance (Å)	PRMT5 C
Asn128(OD1)	3.0	Trp599(NE1)
Asn135 (OD1)	2.8	Thr625(OG1)
Leu191(O)	3.2	Ile621(N)
Trp599(NE)	3.0	Asn128(OD1)
Thr625(OG1)	2.8	Asn135(OD1)

Ile621(N) from chain A is 3.3 Å to Leu191(O) from chain C.

e. PRMT5-MEP50 Interactions

Salt bridges in both A/B and C/D

PRMT5	Distance (Å)	MEP50
Asp161	2.9	Lys191
Glu166	2.6	Arg181
Glu166	2.9	Arg195
Arg44	2.4	Asp89

Salt bridges in either A/B or C/D

PRMT5	Distance (Å)	MEP50
Lys46 (C)	2.9	Glu266 (D)
Lys 86 (C)	2.7	Glu223 (D)
Lys 86 (C)	3.0	Glu178 (D)
His268 (A)	3.5	Asp 115 (B)

Cation-π interactions

PRMT5	MEP50
Arg57	Phe289
Arg63	Trp44

Hydrogen bonds

PRMT5	Distance (Å)	MEP50
Thr19(O)	3.0	Trp44(N)
Leu156(OD1)	2.6	Arg181(NH2)
Leu162(O)	3.0	Ala193(N)
Asn165(OD1)	3.1	Arg195(N)
Arg93(NH2)	2.9	Asn247(OD1)
Arg93(NH2)	3.0	Glu223(O)
Asp160(N)	2.9	Asn154(O)
Asn165(ND2)	2.8	Arg195(N)
Ser65(OG)	3.1	Tyr112(O)

f.

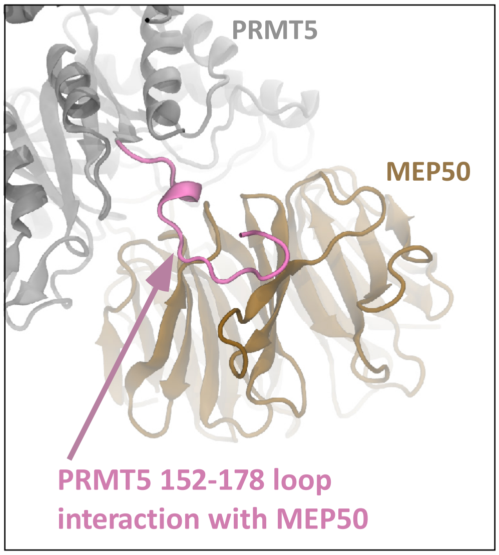

Supplemental Figure S4. PRMT5 interacting residues with SAH, dimer interface, tetramer interface, and with MEP50- A. Residues that we identified in the structure interacting with SAH are shown, with their corresponding hydrogen bonded atom in SAH and the distance in angstroms. * indicates residues that may also be involved in catalysis. B. Ligplot representation of the hydrogen bonding and neighboring residues around SAH in the structure. Inset: SAH pose from 3UA3. C. PRMT5 dimer interface residues, split into salt bridges in the dimerization arm and salt bridges and hydrogen bonds in the N- and C- terminal domains. D. PRMT5 tetramer interface residues, split into salt bridges and hydrogen bonds, with distances listed in angstroms. E. PRMT5 and MEP50 interacting residues are shown, with salt bridges, cation-π interactions and hydrogen bonds illustrated. F. Cartoon representation of the PRMT5 152-178 loop and its interactions with MEP50.
