# Supplementary material for: Structure of the Arginine Methyltransferase PRMT5-MEP50 Reveals a Mechanism for Substrate Specificity
Source: PLoS One. 2013 Feb 25;8(2):e57008. doi: 10.1371/journal.pone.0057008 (PMC3581573; doi:10.1371/journal.pone.0057008)
Supplement: Figure S5 — R3me2s antibody response on peptide array. A high-density peptide array was probed with R3me2s antibody (Millipore #07–947) to measure the baseline signal to determine if neighboring PTMs modulate the response. Peptides are listed in text on the left. In the middle, black boxes represent the presence of a particular modification on a peptide. The histogram on the right shows the relative antibody signal. Pink bar shows the signal on the H4(1–20)R3me2s peptide. Green boxes show the presence of R3me1 or R3me2s. (PDF) [file pone.0057008.s005.pdf]

doi: 10.1371/journal.pone.0057008

H2A/H4R3me2s antibody alone recognition of H4 peptides on JPT Array:

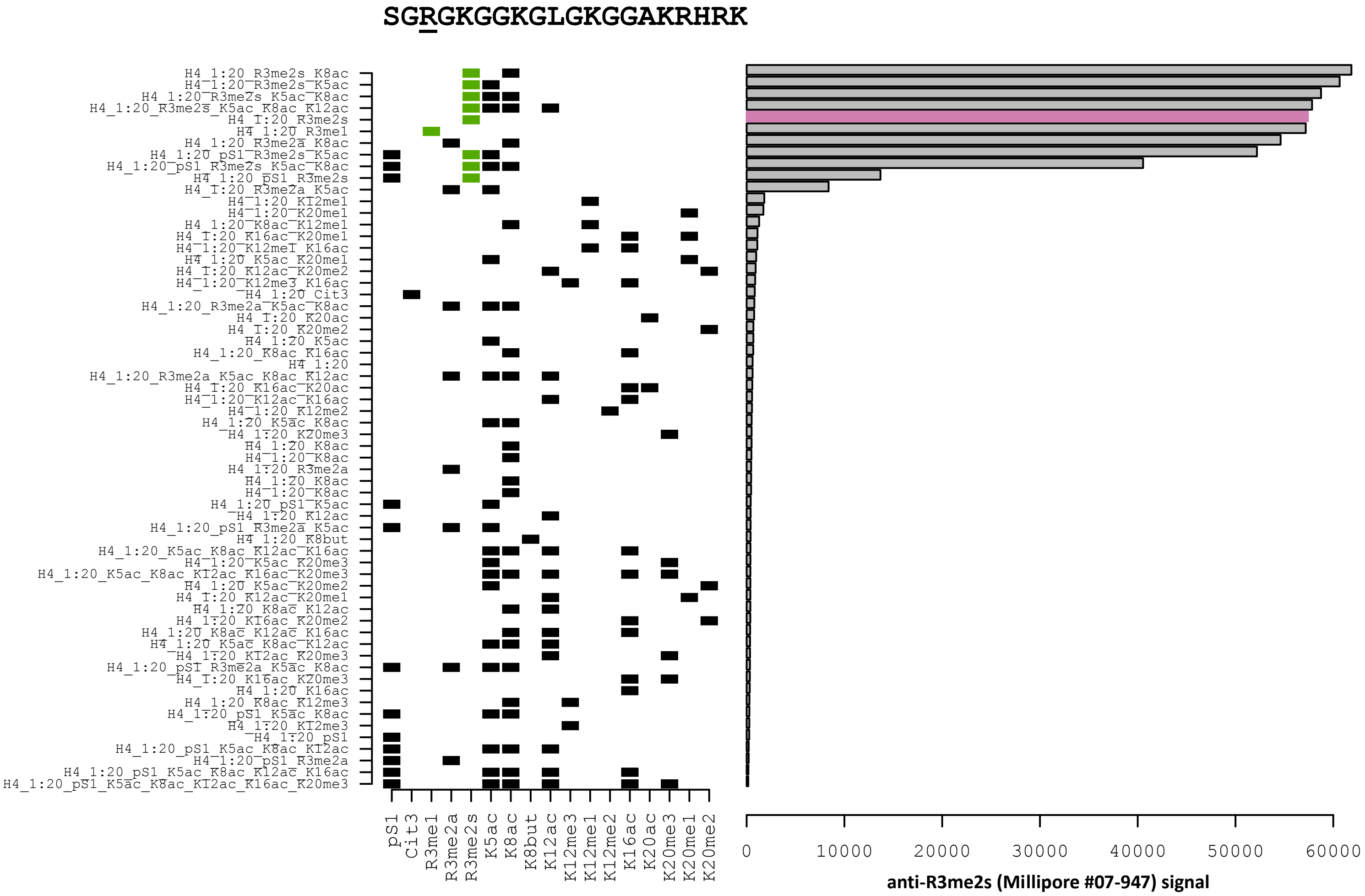

Supplemental Figure S5. R3me2s antibody response on peptide array- A high-density peptide array was probed with R3me2s antibody (Millipore #07-947) to measure the baseline signal to determine if neighboring PTMs modulate the response. Peptides are listed in text on the left. In the middle, black boxes represent the presence of a particular modification on a peptide. The histogram on the right shows the relative antibody signal. Pink bar shows the signal on the H4(1-20)R3me2s peptide. Green boxes show the presence of R3me1 or R3me2s.
