# Supplementary material for: Structure of the Arginine Methyltransferase PRMT5-MEP50 Reveals a Mechanism for Substrate Specificity
Source: PLoS One. 2013 Feb 25;8(2):e57008. doi: 10.1371/journal.pone.0057008 (PMC3581573; doi:10.1371/journal.pone.0057008)
Supplement: Figure S6 — Recombinant human PRMT5 is not complexed with MEP50. A. Recombinant human PRMT5 produced in 293 cells was immunoblotted for PRMT5 (Coomassie stain on left, immunoblot on right). B. Recombinant GST-tagged human MEP50 and human PRMT5 were immunoblotted with an antibody specific for human MEP50 (membrane stain on left, immunoblot on right). (PDF) [file pone.0057008.s006.pdf]

*doi: 10.1371/journal.pone.0057008*

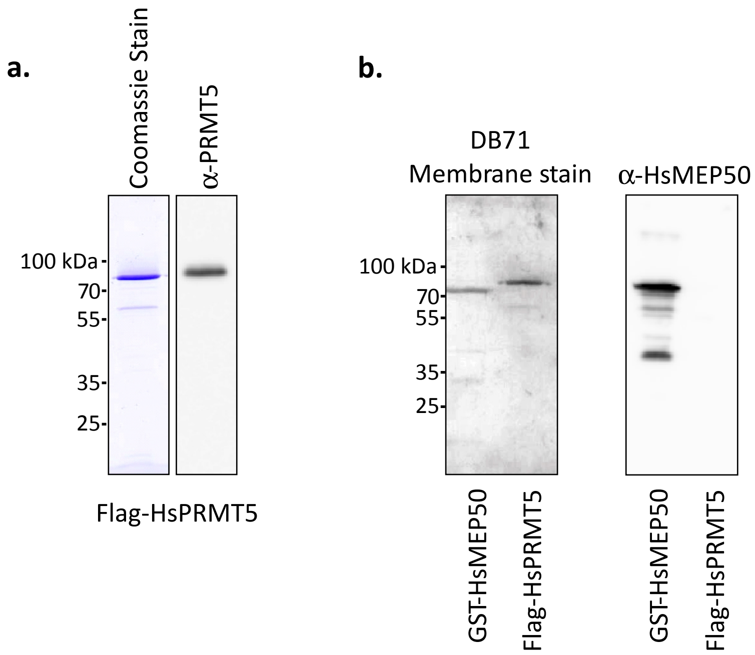


**Supplemental Figure S6. Recombinant human PRMT5 is not complexed with MEP50-** A. Recombinant human PRMT5 produced in 293 cells was immunoblotted for PRMT5 (Coomassie stain on left, immunoblot on right). B. Recombinant GST-tagged human MEP50 and human PRMT5 were immunoblotted with an antibody specific for human MEP50 (membrane stain on left, immunoblot on right).
