# Supplementary material for: Structure of the Arginine Methyltransferase PRMT5-MEP50 Reveals a Mechanism for Substrate Specificity
Source: PLoS One. 2013 Feb 25;8(2):e57008. doi: 10.1371/journal.pone.0057008 (PMC3581573; doi:10.1371/journal.pone.0057008)
Supplement: Figure S7 — Electron microscopy and reconstruction. A. Recombinant class average 2D projections of PRMT5-MEP50. B. PRMT5-MEP50 incubated with recombinant Nucleoplasmin class average 2D projections. The additional density from Nucleoplasmin was observed centered on MEP50 (yellow arrows). C. 3D electron microscopy reconstruction of PRMT5-MEP50 complexed with Nucleoplasmin. PRMT5-MEP50 and Nucleoplasmin molecules from the structure were placed in the density map. The density map is shown wire mesh. (PDF) [file pone.0057008.s007.pdf]

doi: 10.1371/journal.pone.0057008

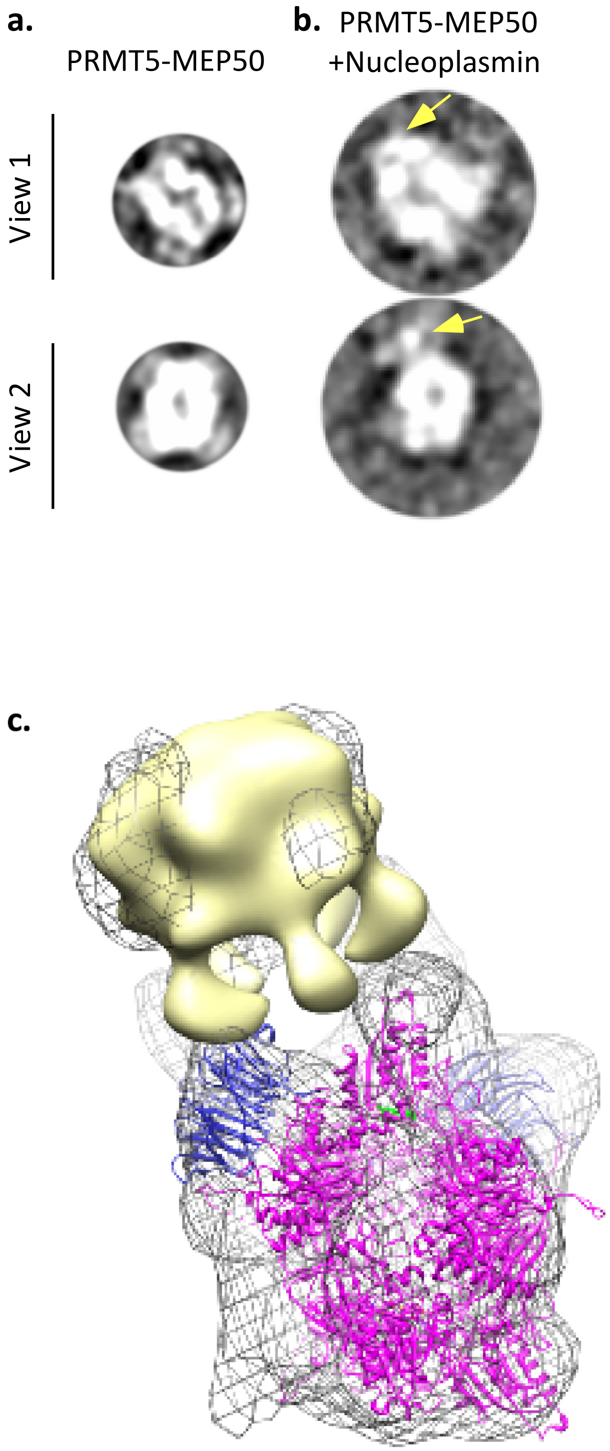

<u>Supplemental Figure S7. Electron microscopy and reconstruction</u> A. Recombinant class average 2D projections of PRMT5-MEP50. B. PRMT5-MEP50 incubated with recombinant Nucleoplasmin class average 2D projections. The additional density from Nucleoplasmin was observed centered on MEP50 (yellow arrows). C. 3D electron microscopy reconstruction of PRMT5-MEP50 complexed with Nucleoplasmin. PRMT5-MEP50 and Nucleoplasmin molecules from the structure were placed in the density map. The density map is shown wire mesh.
