# Supplementary material for: Structure of the Arginine Methyltransferase PRMT5-MEP50 Reveals a Mechanism for Substrate Specificity
Source: PLoS One. 2013 Feb 25;8(2):e57008. doi: 10.1371/journal.pone.0057008 (PMC3581573; doi:10.1371/journal.pone.0057008)
Supplement: Figure S8 — SAH Pose Comparison. A. SAH in Xenopus PRMT5 (PDB:4G56). H-bonds are indicated as dashed yellow lines. The sulfur atom is shown in yellow. B. SAH in human PRMT5 (PDB:4GQB), H4R3 in green, PRMT5 in pink. C. SAH of Xenopus and human PRMT5 overlaid. The aminoethanoic acid in the Xenopus structure clashes with the H4R3 guanidinium position in the human structure. D. SAH of rat PRMT1 (Cyan, PDB:1ORI), human PRMT3 (Yellow, PDB:2FYT), mouse PRMT4 (Gray, PDB:2V74), C. elegans PRMT5 (Pink, PDB:3UA3), and human PRMT5 (Pink, 4GQB). (PDF) [file pone.0057008.s008.pdf]

doi: 10.1371/journal.pone.0057008

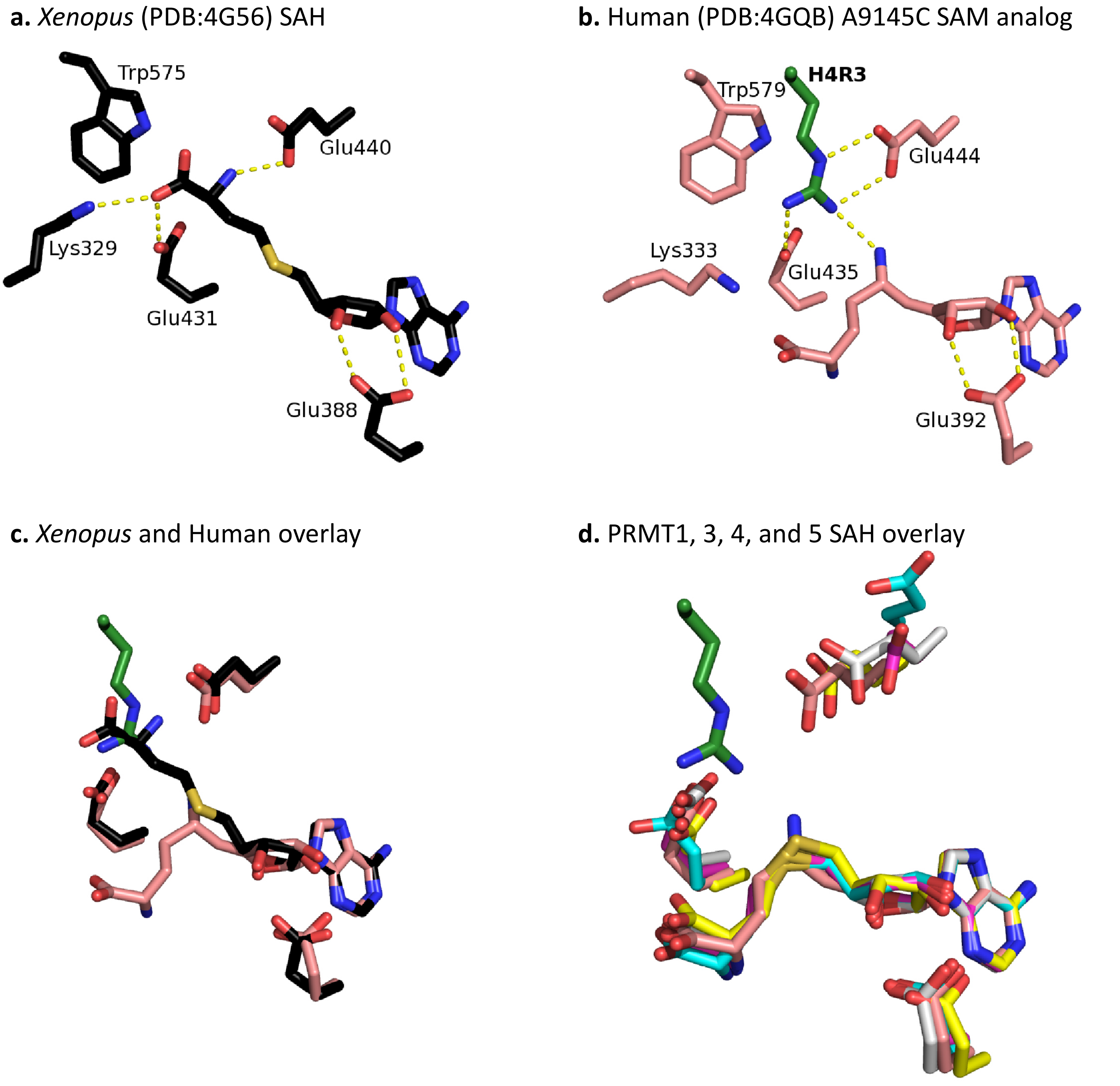

Supplemental Figure S8. SAH Pose Comparison **a.** SAH in *Xenopus* PRMT5 (PDB:4G56). H-bonds are indicated as dashed yellow lines. The sulfur atom is shown in yellow. **b.** SAH in human PRMT5 (PDB:4GQB), H4R3 in green, PRMT5 in pink. **c.** SAH of Xenopus and human PRMT5 overlaid. The aminoethanoic acid in the *Xenopus* structure clashes with the H4R3 guanidinium position in the human structure. **d.** SAH of rat PRMT1 (Cyan, PDB:1ORI), human PRMT3 (Yellow, PDB: 2FYT), mouse PRMT4 (Gray, PDB:2V74), *C. elegans* PRMT5 (Pink, PDB:3UA3), and human PRMT5 (Pink, 4GQB).
